# BRD9 regulates normal human hematopoietic stem cell function and lineage differentiation

**DOI:** 10.1038/s41418-024-01306-5

**Published:** 2024-05-30

**Authors:** Swati Garg, Wei Ni, Basudev Chowdhury, Ellen L. Weisberg, Martin Sattler, James D. Griffin

**Affiliations:** 1 Dana-Farber Cancer Institute, Dept. of Medical Oncology, Boston, MA 02215 USA; 2 Harvard Medical School, Dept. of Medicine, Boston, MA 02215 USA

**Keywords:** Stem-cell research, Cell biology

## Abstract

Bromodomain containing protein 9 (BRD9), a member of the non-canonical BRG1/BRM-associated factor (ncBAF) chromatin remodeling complex, has been implicated as a synthetic lethal target in AML but its function in normal human hematopoiesis is unknown. In hematopoietic stem and progenitor cells (HSPC) genomic or chemical inhibition of BRD9 led to a proliferative disadvantage and loss of stem cells in vitro. Human HSPCs with reduced BRD9 protein levels produced lower numbers of immature mixed multipotent GEMM colonies in semi-solid media. In lineage-promoting culture conditions, cells with reduced BRD9 levels failed to differentiate into the megakaryocytic lineage and showed delayed differentiation into erythroid cells but enhanced terminal myeloid differentiation. HSPCs with BRD9 knock down (KD) had reduced long-term multilineage engraftment in a xenotransplantation assay. An increased number of downregulated genes in RNAseq analysis after BRD9 KD coupled with a gain in chromatin accessibility at the promoters of several repressive transcription factors (TF) suggest that BRD9 functions in the maintenance of active transcription during HSC differentiation. In particular, the hematopoietic master regulator GATA1 was identified as one of the core TFs regulating the gene networks modulated by BRD9 loss in HSPCs. BRD9 inhibition reduced a GATA1-luciferase reporter signal, further suggesting a role for BRD9 in regulating GATA1 activity. BRD9 is therefore an additional example of epigenetic regulation of human hematopoiesis.

## Introduction

Hematopoiesis is an elegantly complex process where slowly cycling, self-renewing hematopoietic stem cells (HSC) give rise to a much larger number of mature, differentiated cells while maintaining the stem cell pool. Classically, the process of blood formation is described as a multi-step hierarchical process where undifferentiated, quiescent stem cells differentiate into oligo, multi and uni-potent progenitors that pass through commitment branch points [1–5]. Recently, through the advances of single cell “omics” techniques, a continuous model of hematopoiesis is supported where lineage restricted cells arise from a continuum of low-primed undifferentiated clones of HSC [6–10]. In either model, functionally or phenotypically defined long-term hematopoietic stem cells, and lineage primed-committed cells remain transcriptionally different entities [11].

The daily requirement of replenishing large numbers of differentiated functional blood cells from a relatively miniscule stem cell population demands a high degree of transcriptional plasticity with dynamic regulation in response to intrinsic and extrinsic signals. Epigenetic regulators are often used to relay such context dependent changes without altering the underlying genetic information. Nucleosome remodeling is one of the mechanisms of epigenetic regulation where enzymes regulate DNA accessibility by adding or deleting chemical modifications on histones in the nucleosome [12]. Four major subfamilies of ATP dependent enzymes bring about nucleosomal remodeling, these are called: Chromodomain Helicase DNA-binding (CHD/NuRD/Mi-2), Imitation Switch (ISWI), INO80, and mammalian Switch Sucrose Non Fermentable (mSWI/SNF) [13]. mSWI/SNF is primarily responsible for sliding the nucleosome along the DNA and thereby directly regulating access to the DNA binding factors. The mSWI/SNF complexes are known to participate in many different cell fate determinations including hematopoiesis [14, 15]

mSWI/SNF, also known as the BRG1 associated factor (BAF) complex family, consists of twenty nine known members which are assembled into three subfamilies of complexes, canonical BAF (cBAF), polybromo-associated (PBAF) and the recently discovered GLTSCR1-containing GBAF, also known as non-canonical BAF (ncBAF) [16–18]. Each complex consists of about 10–15 subunits and a core ATPase motor which controls the sliding. In addition, each complex contains components with domains that determine the local recognition, interaction and positioning along the nucleosome through domains such as AT-rich domain (ARID), plant homology finger domain (PHD), and bromodomains (BRD) [14, 19]. Bromodomains are structurally conserved modules which interact with acetylated lysine residues on proteins and act as histone readers [20]. Detailed knowledge of the interaction between bromodomains and histones has been exploited in pharmacological development of small molecule inhibitors of BRD containing proteins with significant implication as therapeutics for specific diseases [21, 22]. Each of the three types of mSWI/SNF complexes have one or more subunits with a bromodomain, including BRG1, PBRM1, BRM, BRD7 and BRD9. BRD9 is unique to the ncBAF complex and its genetic and pharmacological inhibition is reported to induce synthetic lethality in several cancers, including acute myeloid leukemia (AML) [23–26], synovial sarcoma, non-small cell lung cancer and prostate cancer [27–29]. Reports have also shown the potential of BRD9 targeting in inflammatory disorders and in enhancing anti-tumor immunity [30–32]. In myelodysplasias (MDS) and uveal melanomas (UVM) with mutant SF3B1, BRD9 is reported to undergo alternative splicing leading to inclusion of a poison exon causing mRNA degradation, also implicating BRD9 as a potential oncogene in those tumors [33].

While mutations of SWI/SNF components are observed in cancers, the mutation frequency in hematological malignancies, in particular of BRD9, is rare compared to solid tumors [34]. Interestingly, BRD9 has recently been described as an essential gene in AML, where inhibitors, degraders, or knockdown of BRD9 leads to myeloid differentiation, reduced proliferation, and cell death in murine and human model systems of AML [23, 24, 26, 35], suggesting BRD9 might be a useful therapeutic target in AML patients. Despite these reports of involvement of BRD9 in AML and a recent study of BRD9 in murine hematopoietic model [36], a clear understanding of BRD9 function and its molecular involvement in normal human hematopoiesis has been missing. Here, we report a systematic evaluation of BRD9 function in human CD34+ hematopoietic stem and progenitor cells using genetic and pharmacological inhibition.

## Methods

(See supplementary methods for details)

### Cells, culture and compounds

Cryopreserved primary CD34+ cells derived from mixed donor cord blood units (#70008) and G-CSF mobilized peripheral blood (#70060) were purchased from Stem Cell Technologies. Cells were maintained in the optimal medium containing IMDM (Thermo #12440053) and 20% BIT serum substitute (Stem Cell Technologies #9500), supplemented with cytokines purchased from Miltenyi at a final concentration of 100 ng/mL rh-SCF (#130-096-695), 100 ng/mL rh-FL(#130-096-479), 50 ng/mL rh-TPO(#130-095-752), 100 µM β-mercaptoethanol (Gibco #21985023), 50 ng/mL gentamicin (Gibco #15750060), 10 ng/mL ciprofloxacin (Sigma #73832-100MG), and 35 nM UM171(Xcess Bio #M60223-2S) as published previously [37]. Cells were directly FACS sorted (fluorescent-activated cell sorting) into pre-aliquoted methylcellulose media purchased from Stem Cell Technologies (#4034), supplemented with 50 ng/mL gentamicin and 10 ng/mL ciprofloxacin, and placed in humidified chambers for 10-12 days before manual colony counting.

Effects on megakaryocytic, erythroid or myeloid populations were measured after culture in the defined media known as the HemaTox Megakaryocytic Kit (# 09707), HemaTox Erythroid Cell Kit (#9701) and HemaTox Myeloid Cell Kit (#9704) from Stem Cells Technologies. Freshly thawed cells were treated with BRD9 degraders or transduced cells were FACS sorted directly in these media conditions to examine effects on lineage selection. The BRD9 chemical degrader dBRD9A was purchased from Tocris (#6943/5), and the BRD9 degrader QA68 was a kind gift from Novartis [26].

### Lentiviral vectors

Short hairpin RNA vectors in pLKO.1 in the puromycin backbone were purchased from Sigma and puromycin sequence was replaced with GFP in shBRD9: TRCN00001283**33**, TRCN00001276**34**, TRCN00001277**80**, and TRCN00001310**81** and control shRNA vector SHC002 using BamHI (Thermo #FD0054), KpnI (Thermo#FD0524), and T4 ligation (Thermo #EL0011). BRD9 hairpins are denoted as the last two digits of their catalog number throughout this manuscript. BRD9 cDNA vector was purchased from GeneCopoeia (#H9078) and the open reading frame (ORF) was custom cloned behind EF1ɑ promoter in pLVX-EF1α-IRES-mCherry Vector (Takara#631987) with a C-terminal HA and 3xFlag tag. Target regions for each hairpin were identified in the BRD9 wild-type cDNA overexpression (OE) vector and site-directed silent mutations were introduced at the target site of shBRD9#81 as per the recommendations [38]. An ORF clone for GATA1(#OHu22697) was purchased and custom cloned in pLVX-EF1α-IRES-mCherry vector using GenScript reagent services. Lentiviral transduction of primary cells was performed as per the recommendations [39] (see supplementary methods for details).

### Mouse experiment and ethical approval

Six weeks old female NBSGW mice (NOD.Cg-*Kit*^*W-41J*^ *Tyr* ^+^ *Prkdc*^*scid*^ *Il2rg*^*tm1Wjl*^/ThomJ) were purchased from The Jackson Laboratory (RRID: IMSR_JAX:026622) and housed at the DFCI animal resource facility for two weeks before the start of experiments. Procedures were approved by the DFCI Institutional Animal Care and Use Committee (IACUC) under protocol 16-009. Equal numbers (60,000) of lentiviral transduced, unsorted (GFP+/–) human CD34 cells were injected via tail vein into 6–7 mice each group (control and 2 hairpins against BRD9). Femoral bone marrow aspiration was performed after cell injection at week 6 and week 10 under general anesthesia and mice were sacrificed at week 16 for collection of spleen and long bones for marrow extraction.

### Bioinformatics analysis

Publicly available RNAseq datasets were downloaded through iDEP [40]. Gene ontology enrichment was done using ShinyGo [41] and GSEA desktop application [42, 43]. Differential gene expression analysis on RNAseq data was performed using DeSeq2 [44]. Heatmaps from the normalized basemean counts were generated using iDEP keeping Euclidean distance, average correlation and a z-score cut off to 3 for hierarchical clustering. Transcription factor and cofactor annotations were done through the published gene lists at human transcription factor database [45] and transcription cofactor database [46]. The assay of transposase accessible chromatin with sequencing (ATACseq) data was analyzed using the DiffBind package [47]. Transcription factor enrichment analysis on differential peaks was done using Chip-Atlas [48, 49]. Publicly available chromatin immunoprecipitation (ChIP) seq data were downloaded from the Gene Expression Omnibus (GEO) [50] and ENCODE project [51]. ChIP tracks were visualized and overlay plots were prepared using Integrated Genome Viewer (IGV) [52]. Experiment layout designs were created using BioRender.com.

### Statistics

GraphPad prism was used for all the statistical analysis and graph preparation. Unpaired, two tailed t-tests were performed for comparison of two groups. For comparing differences between multiple groups at a single time point ordinary one-way ANOVA with multiple comparison to the reference group and Dunnett’s correction was used. Multiple conditions between two groups and multiple groups at more than one time point were compared using ordinary ANOVA with Fisher’s LSD test and ordinary ANOVA with Tukey’s multiple comparison test. Significant differences were shown in the graphs between control and test condition only. Numbers of asterisks correspond to the decimal zeros in front of the p value (*<0.05, **<0.005, ***<0.0005 and ****<0.00005). Typical experiments are shown, unless otherwise indicated. Each experiment was performed minimum 2 times independently and each biological replicate had minimum 3 to maximum 18 technical replicates per condition. Data normality test, variance, statistics test used and number of biological replicates per experiment per data plot are listed in the respective figure legend.

## Results

### BRD9 regulates proliferation and differentiation of human HSPCs in vitro

**Fig. 1:**
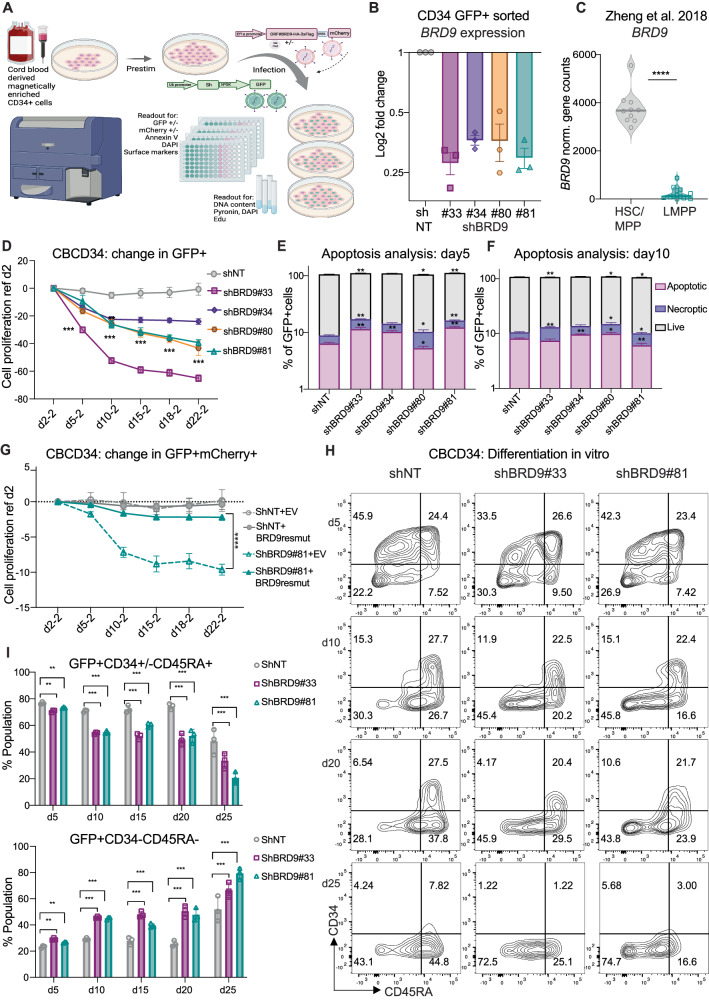
BRD9 regulates HSPC proliferation, survival and differentiation in vitro. **A** Experimental design for lentiviral infection in HSPC and related in vitro experiments. **B** *BRD9* knockdown level in HSPCs infected with shBRD9 compared to control (mean and standard error of mean (SEM) are plotted for *n* = 3 independent experiments). **C** Expression of *BRD9* in hematopoietic stem cells (HSC) and multipotent progenitors (MPP) compared to lympho-myelo primed progenitors (LMPP) in published dataset GSE97104 (violin plots sowing individual data point and median; *p* value < 0.0001 in unpaired t-test). **D** proliferation of shBRD9 and control GFP+ HSPCs in culture over a period of time starting day 2 post infection, negative change refers to proliferative disadvantage while no change depicts no significant difference in GFP+ fraction (mean and standard deviation (SD) is plotted for one representative experiment out of *n* = 4 independent experiments; *p* value < 0.005 in unpaired t-test compared to control at each time point). **E**, **F** Flow cytometric analysis of apoptosis using annexin V and DAPI in GFP+ HSPCs on day 5 and day 10 post infection (mean and SEM is plotted for one representative experiment of *n* = 4 biological replicates; *p* value < 0.005 in unpaired t-test compared to control at each time point). **G** Proliferation rescue in HSPC by ectopic expression of hairpin resistant mutant BRD9 in shRNA (GFP) and ORF (mCherry) co infected cells (*n* = 3, representative plot showing mean and SD at each time point for 6 technical replicates, unpaired t-test between shBRD9#81+resmut BRD9 over-expression vector and shBRD9#81+ empty vector resulted in *p* value < 0.0001). **H** Representative flow cytometry profile of CD34 and CD45RA surface expression on GFP+ HSPCs in control and BRD9 shRNA (rows) on days post infection (column) show (**I**) gradual loss of CD34+CD45RA+ and increase in differentiated CD34–CD45RA– fraction (*n* = 5; mean and SD are plotted along with individual data point; *p*-values resulting from unpaired t-test between shNT control and shBRD9 conditions is shown as *<0.05, **<0.005, ***<0.0005 and ****<0.00005, also see methods section).

To identify the function of BRD9 in the human hematopoietic system, we used small hairpin (sh) RNA mediated knock down (KD) or chemical degraders targeting BRD9 to suppress its expression in cord blood (CB) and mobilized peripheral blood (mPB) derived human CD34+ cells. Cells were infected with GFP tagged BRD9 targeting shRNA vectors or controls and analyzed by flow cytometry for proliferation, apoptosis, cell cycle analysis, and differentiation (Fig. 1A). The efficacy of BRD9 shRNA to reduce BRD9 in GFP sorted CD34 cells was confirmed at the level of both mRNA and protein (Fig. 1B and Supplementary Fig. 1A), and a hairpin-resistant mutant of BRD9 cDNA vector was used as a negative control (Supplementary Fig. 1B). Using the publicly available RNAseq data from GSE115798 and GSE97104, we observed a higher mRNA expression of BRD9 in HSC compared to lineage primed cells (Fig. 1C, Supplementary Fig. 1C). We found that in our optimal suspension culture medium containing SCF, FL, TPO and UM171, the expression level of BRD9 mRNA goes down in CD34 cells with increasing time in culture (Supplementary Fig. 1D), suggesting that BRD9 expression is correlated with an undifferentiated state and/or stemness. In suspension cultures, a moderate proliferative disadvantage was observed for CD34+ cells with BRD9 KD in multiple independent experiments (Fig. 1D). Parallel apoptosis analysis using annexin V and DAPI staining showed a slight induction of apoptosis, in line with the observed loss of cells in culture (Fig. 1E, F). DNA content analysis using pyronin gamma and DAPI suggested a modest increase of the G0-subG1 subpopulation in cells infected with hairpins against BRD9 (Supplementary Fig. 1E, F). To rule out the possibility of off-target effects of the hairpins, we measured the proliferative capacity of GFP+ (hairpin) mCherry+ (ORF) cells over time which were co-infected with the most efficient hairpin (sh#81) and BRD9 over-expression mutant vector resistant to this hairpin (resmut). The proliferative disadvantage caused by shBRD9 was partially reversed by expression of the resistant mutant in both CBCD34 and MOLM14 AML cells suggesting a gene specific phenotype (Fig. 1G, Supplementary Fig. 1G). Next, we asked if BRD9 plays a role in differentiation of CD34 cells by staining cells with CD34 and CD45RA at multiple time points. We observed a significant gain in the CD34 negative, CD45RA negative, (CD34–CD45RA–) differentiated population after BRD9 KD (Fig. 1H–I, Supplementary Fig. 1H). Treatment of CD34 cells with the BRD9 degrader dBRD9A showed a similar gain of differentiated cells in a concentration dependent manner (Supplementary Fig. 1H). These data suggest that BRD9 is essential to maintain the stemness of human hematopoietic stem cells in short term cultures.

### BRD9 plays a role in hematopoietic lineage selection and is indispensable for megakaryocytic differentiation

**Fig. 2:**
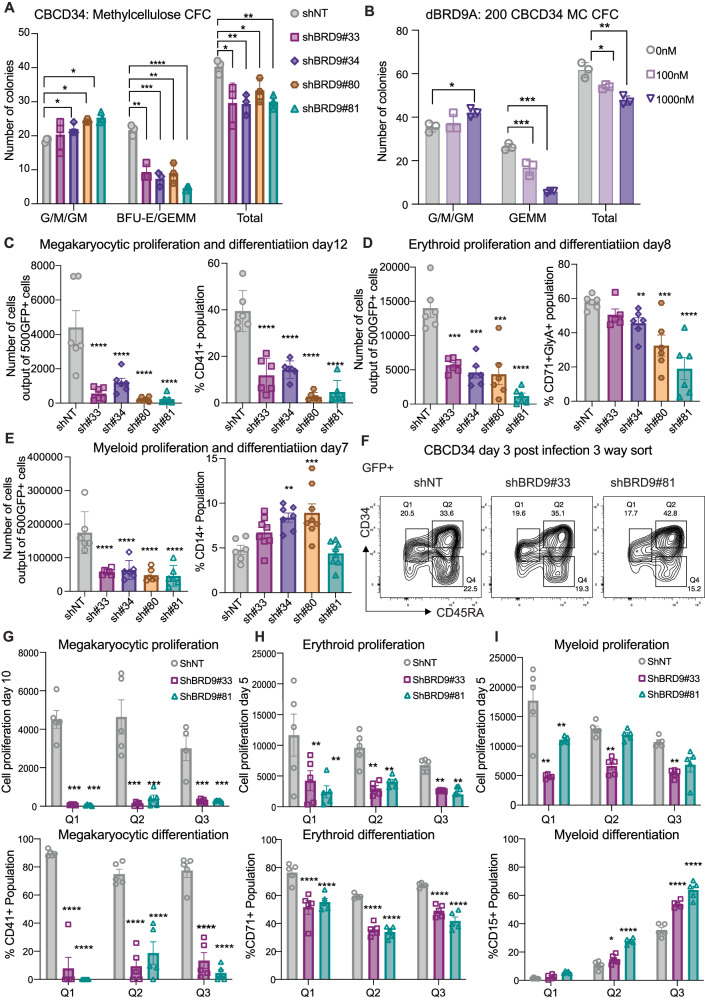
HSPC multilineage potential and lineage differentiation is regulated by BRD9. **A**, **B** Methylcellulose colony formation capacity of HSPCs infected with control or BRD9 shRNA (*n* = 4) and in presence of DMSO or increased concentrations of dBRD9A (*n* = 6) (plots show individual data points with mean and SD; and *p* values from unpaired t-tests compared to control from one biological replicate). HSPC proliferation and differentiation in megakaryocytic (**C**), erythroid (**D**), and myeloid media (**E**) (all technical replicates with their mean and SEM from one of the *n* = 5 independent experiments are plotted and *p* values from unpaired t-test between shNT and BRD9 shRNA conditions are shown). **F** FACS sort gating strategy for GFP+ cells in shNT and shBRD9 infected cells using CD34 and CD45RA along differentiation trajectory, where Q1 depicts the least and Q3 the most differentiated state. Cell proliferation and differentiation in **G** megakaryocytic lineage promoting media showing cell count and CD41 expression, **H** erythroid media with cell counts and CD71 expression and **I** myeloid media with relative cell proliferation and CD15 surface expression from sorted fractions (plots show individual data points, mean and SEM and *p* values from unpaired t-tests compared to control as *<0.05, **<0.005, ***<0.0005 and ****<0.00005, also see methods section) .

Differentiation of HSCs into one of the several blood cell types is dictated by cell extrinsic and intrinsic factors. Well established combinations of cytokines are known to promote myeloid, erythroid and megakaryocytic specific differentiation of HSPCs in vitro. After observing the proliferative loss and enhanced differentiation of HSPCs with BRD9 KD, we asked if BRD9 regulates lineage selection in short-term cultures. First, we performed the classical methylcellulose colony formation assay using BRD9 shRNA and two different chemical degraders dBRD9A and QA68. In several independent experiments, BRD9 KD cells had significantly reduced capacity to give rise to GEMM multi-lineage mixed colonies (granulocyte, erythrocyte, monocyte, megakaryocyte) compared to control, accompanied by a slight increase in myeloid CFU-G/M (granulocyte-macrophage) colonies (Fig. 2A, Supplementary Fig. 2A). A similar effect on colony forming capacity was seen in the presence of two different BRD9 degraders in a concentration dependent manner (Fig. 2B, Supplementary Fig. 2B).

We then asked if BRD9 plays a role in lineage selection during short term cultures by measuring cell proliferation and lineage specific marker expression as a surrogate in specialized HemaTox media for megakaryocytic, erythroid, and myeloid differentiation. We observed significant loss of proliferation and differentiation to cells expressing the megakaryocyte marker CD41 following treatment with either BRD9 shRNA or dBRD9A (Fig. 2C, Supplementary Fig. 2C). Similarly, cells placed in erythroid-priming medium showed a moderate proliferative disadvantage and delayed and reduced erythroid differentiation as measured by CD71+CD235a+ expression after BRD9 downregulation (Fig. 2D, Supplementary Fig. 2D). In myeloid differentiation medium, cells with BRD9 downregulation showed a mild proliferative decrease coupled with rapid differentiation to CD15+ cells (data not shown) or CD14+ cells (Fig. 2E, Supplementary Fig. 2E).

In order to determine if alterations in lineage differentiation arose from the most primitive HSPCs in culture, we used CD45RA [53] in combination with CD34 and sorted the infected GFP+ cells into three fractions with increasing differentiation potential: The most primitive cells (Q1) were defined by the immunophenotype CD34+CD45RA–, intermediate cells (Q2) were co-positive for CD34+CD45RA+, slightly more committed cells were in Q3 were defined as expressing CD34–CD45RA+, while the most differentiated cells were co-negative CD34–CD45RA– (Fig. 2F). Notably, the megakaryocytic and erythroid differentiation potential of control cells dropped from Q1 to Q3 whereas cells with the highest myeloid potential were in the CD34–CD45RA+ Q3 population. We observed that proliferation and differentiation into the megakaryocytic lineage was severely diminished in BRD9 KD cells regardless of their priming along the CD34, CD45RA trajectory (Fig. 2G). In erythrocytic culture conditions, the disadvantage in proliferation and differentiation in the presence of BRD9 shRNA compared to control was most marked in cells from Q1 fraction compared to cells from Q2 and Q3 fractions, suggesting a more prominent effect of BRD9 depletion on the erythroid differentiation capability of the most immature CD34+CD45RA– population (Fig. 2H). Lastly, the difference in myeloid proliferation was significant only in the immature Q1 population. However, the more differentiated Q3 population acquired CD15+ more rapidly in BRD9 KD cells indicating a significant acceleration of terminal differentiation by BRD9 inhibition in myeloid-primed cells (Fig. 2I).

These observations suggest the importance of BRD9 in regulating lineage determination of hematopoietic stem cells where the loss of BRD9 leads to enhanced commitment to myeloid cells at the expense of megakaryocytic and erythroid differentiation. BRD9 loss severely abrogates commitment to megakaryocytic lineage differentiation, suggesting a unique and critical dependency of the megakaryocytic lineage on BRD9.

### Long term (LT)-HSC potential is affected by the loss of BRD9 expression

**Fig. 3:**
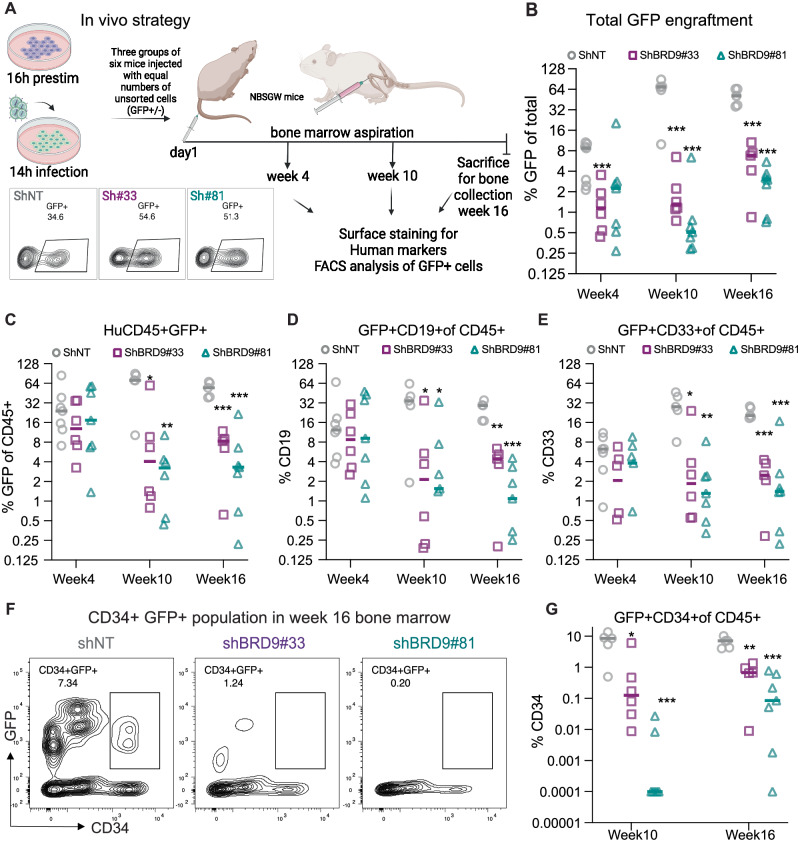
Long term multilineage potential of human HSPCs in immunocompromised mice is affected after BRD9 KD. **A** Experimental layout and GFP+ fraction of control and BRD9 shRNA infected cells (60,000 cells (unsorted for GFP) per mouse was injected on day1 post infection in 7 mice for control and 6 mice each for the two BRD9 hairpins). **B** GFP+ engraftment from HSPCs in total marrow cells at short-, intermediate- and long-term (week 4, 10 and 16) post-transplant. **C** GFP+ of human CD45+ engraftment in the mice marrow. **D** CD19 + B lymphoid and **E** CD33+ myeloid lineage engraftment from GFP+ HSPCs. **F**, **G** Flow cytometry profile of GFP and CD34 expression in bone marrow of one representative mouse per group and CD34 output from engrafted GFP+ HSPCs in week 10 and week 16 bone marrow. Each mouse is plotted along with the calculated median per group as the horizontal line; y-axis is in the log-scale to accommodate all the values; difference between control and BRD9 KD conditions is calculated by unpaired t-test and the significance is shown by depiction of *p* values (see methods section).

Functional read out of long-term hematopoietic stem cell potential (LT-HSC) is classically done by injecting limiting cell numbers in sub-lethally irradiated mice. To this end, we injected equal numbers of lentivirally transduced (unsorted for GFP) CBCD34 cells into the tail vein of severely immunocompromised, non-irradiated NBSGW female mice and checked the engraftment of human HSPCs in the bone marrow at weeks 4 and 10 and in total marrow and spleen at week 16 post-injection (Fig. 3A). Engraftment of human CD45+ cells and GFP+ cells at week 16 in bone marrow in one representative mouse per group is shown in Sup. Fig. 3A. Engraftment of GFP+ cells at week 4 was slightly lower following BRD9 KD and engraftment dropped further at week 10 and week 16 (Fig. 3B), while the overall CD45+ engraftment (GFP+/–) in all conditions remained similar (Supplementary Fig. 3B). Cells staining double positive for human CD45 and GFP showed a similar trend as that of total GFP+ cells, indicating that the GFP signal was indeed from the engrafted human CD34+ cells (Fig. 3C). No preferential bias was seen in the engraftment of lymphoid or myeloid lineage cells marked by CD19 and CD33 respectively and calculated as a ratio of lymphoid to myeloid cells (Supplementary Fig. 3C). Surface staining revealed a significant reduction of both lymphoid and myeloid cells in the engrafted BRD9 KD cells (Fig. 3D, E). A loss of CD41+ megakaryocytic and CD71+ erythroid lineage cells was observed in mice injected with BRD9 KD cells compared to the control (Supplementary Fig. 3D, E). Moreover, the fraction of human CD34+ cells in the marrow of NBSGW mice injected with BRD9 KD cells was significantly reduced compared to that in shNT control mice at week 10 and 16 (Fig. 3F, G). Taken together these data suggest a role of BRD9 in the pan-lineage long-term repopulation potential of human HSPCs.

### BRD9 is important to maintain regulatory gene networks associated with stemness

**Fig. 4:**
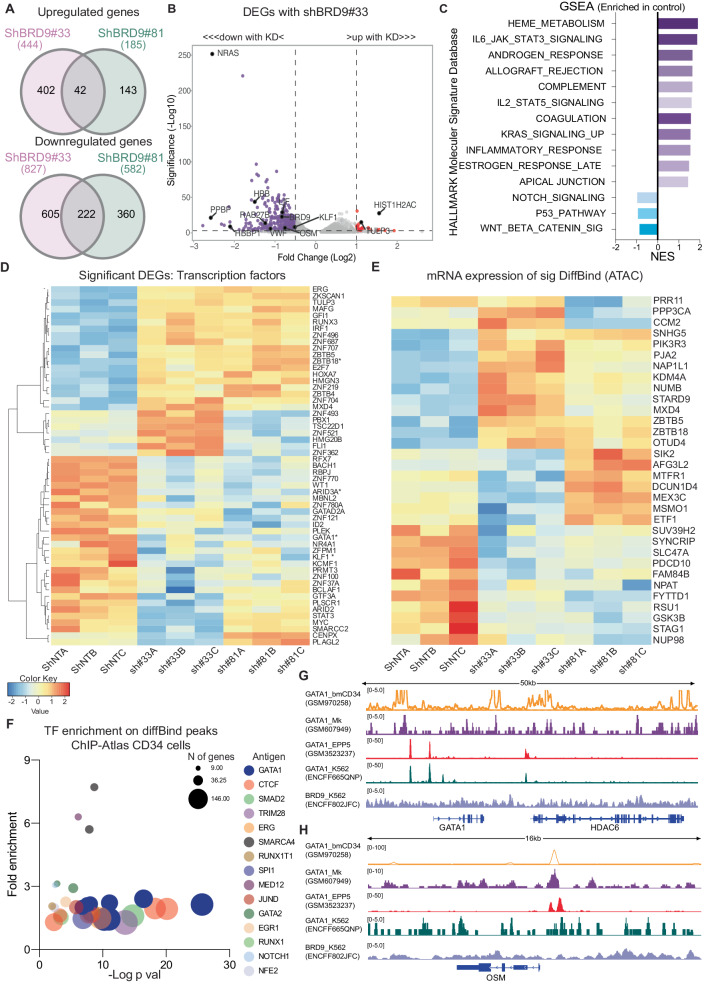
Differential gene expression and chromatin accessibility analysis after BRD9 KD in HSPCs. **A** Common up and down regulated genes with two different shRNA against BRD9 compared to control. **B** Volcano plot showing differentially expressed genes (DEG) in shBRD9#33 where significance threshold for a DEG is kept at the padj 0.01 level, downregulated genes cut off at log2 fold change of ≤–0.5 and positive fold change at ≥1. **C** Gene set enrichment analysis against hallmark molecular signature database on common DEGs in two hairpins. **D** Heatmap showing expression level of significantly different transcription factor genes (DESeq2 padj ≤0.01; basemean values plotted with correlation distance and average linkage). **E** Heatmap showing mRNA expression of genes which showed significantly different chromatin accessibility in ATACseq DiffBind analysis. **F** ChIP Atlas TF enrichment analysis filtered on CD34 HSC cell type in blood tissue type on gained accessibility ATACseq peaks, plot showing significance level on x-axis and fold enrichment on y-axis. **G**, **H** Integrated genome browser (IGV) images for GATA1 and BRD9 ChIP overlay peaks (data scaling shown in the parenthesis) in different datasets for bone marrow derived CD34 cells, megakaryocytic cells, CD34 derived early erythroid progenitors and erythroleukemia K562 cell line at *GATA1* and *OSM*.

After observing the role of BRD9 in HSC fate, we looked for possible molecular mechanisms. To this end, we performed RNAseq and ATACseq on GFP sorted CBCD34 cells, 72 h post shRNA infection (Supplementary Fig. 4A). Significant overlap was seen between the two hairpins used against BRD9 during the analysis of differentially expressed genes (DEG) from non-target control (Fig. 4A). A generalized transcriptional repression was observed after BRD9 KD, since the number of downregulated genes (*p*adj ≤ 0.05, log2FC ≤ –0.5) was almost five times more than the number of upregulated genes (*p*adj ≤ 0.05, log2FC ≥ 0.5). Along with *BRD9* itself, many important genes involved in megakaryocytic and erythroid lineage function such as *VWF*, *RAB27B*, *PPBP*, and *KLF1* [54, 55] were among the top downregulated genes as shown in the volcano plots with the two hairpins (Fig. 4B, Supplementary Fig. 4B). Gene Set Enrichment Analysis (GSEA) on hallmark molecular signature database (msigDB) showed genes involved in heme metabolism, coagulation, and JAK-STAT3 signaling were significantly enriched in cells infected with non-target control compared to BRD9 KD cells (Fig. 4C). Transcription factor (TF) enrichment analysis using ChEA and ENCODE suggested GATA1 and GATA2 as the most significant TFs that are likely involved in the regulation of expression of genes which were downregulated with BRD9 KD in CD34 cells (Supplementary Fig. 4C).

Since BRD9 is a member of ncBAF complex, a known chromatin modulator, we queried the human Transcription-Factor-Database (TFDB) and found that of the 1213 TFs sequenced in our dataset, 102 were significantly differentially expressed genes (DEGs) in BRD9 KD cells compared to control (padj≤0.05, common in two hairpins against *BRD9*). Hematopoietic master regulators including *GATA1*, the megakaryocytic TF *ARID3A*, and the erythroid TFs *KLF1* and *FOG1* were among the genes with reduced expression in CBCD34 cells after BRD9 KD, whereas *TULP3*, *HOXA7* and *ZBTB4*, *ZBTB5* and *ZBTB18* were among the TFs with increased expression (Fig. 4D). We also queried the TF cofactor (TcoF) DB and identified 118 TcoF as significant DEGs (*p*adj ≤ 0.05 in 2shs) out of 933 TcoF sequenced in our dataset (Supplementary Fig. 4D). While *SETD7* and *RNF2* were among the most interesting downregulated TcoF, we did note a slight increase in the expression of *CDKN1B* which could contribute to the proliferation loss upon BRD9 KD in these cells. To understand better if the loss of BRD9 KD cells in long term in vivo assays was indeed accompanied by a loss of an HSC gene signature, we queried our data for the list of HSC associated genes described in the literature [56], and found no gain of any HSC associated signature in BRD9 KD cells. A similar bioinformatics analysis for other BAF members revealed no significant changes in their expression levels after BRD9 KD.

Notably, despite the significant changes in the gene expression levels in RNAseq data, we observed only very subtle changes in overall chromatin accessibility after BRD9 KD. The DiffBind analysis revealed 163 differentially accessible, gained peaks in cells treated with either of the two hairpins against BRD9 compared to the control. When the annotated peaks were subjected to Gene Ontology (GO) enrichment, the most significantly enriched biological processes were negative regulation of macromolecule biosynthetic processes and negative regulation of transcription (Supplementary Fig. 4E). These GO terms are consistent with the observation of more downregulated genes after BRD9 KD than upregulated genes in DEG analysis of RNAseq data. Next, we examined our RNAseq data for expression of the increased accessibility genes in ATACseq and found that genes like *ZBTB5*, *ZBTB18* and *MXD4* were among the top candidates, where gain in accessibility (ATACseq) correlated with an increased transcription level of each gene (RNAseq) (Fig. 4E). These genes code for TFs which are known transcriptional suppressors. We then subjected differentially accessible genes for TF enrichment using ChIP-Atlas data filtered on CD34 hematopoietic cells and found GATA1 to be the most significantly enriched TF involved in regulation of these genes (Fig. 4F).

Finally, we asked if GATA1 indeed regulates the DEGs observed after BRD9 KD in CD34 cells and whether BRD9 shows any experimental evidence of chromatin interaction in those regions. To this end, we downloaded publicly available data from Gene Expression Omnibus (GEO) and ENCODE and overlaid the chromatin immunoprecipitation (ChIP) tracks using IGV. As expected [57], GATA1 binds near its own promoter in CD34+, megakaryocytes, erythroid progenitors, and the erythroleukemia cell line K562, which showed a generalized signal for BRD9 in this region (Fig. 4G). Of note, Oncostatin-M (*OSM*) one of the most significantly downregulated genes after shRNA or degrader mediated downregulation of BRD9 may be regulated by GATA1 in CD34, megakaryocyte and erythroid cells (Fig. 4H). Lineage specific regulation of the erythroid TF *KLF1* and megakaryocytic TF *MXD3* by GATA1 and enhanced chromatin interaction of BRD9 was observed (Supplementary Fig. 4F, G). Interestingly, the regions regulated by GATA1 in more than one lineage showed an increased permissiveness of BRD9 interaction with chromatin in regions such as the hemoglobin cluster (Supplementary Fig. 4H). However, where GATA1 showed limited regulation, such as in the HOXB cluster in erythroid progenitors, no significant enrichment of the BRD9 ChIP signal was observed (Supplementary Fig. 4I). Taken together, these data suggest a role of BRD9 in maintenance of a gene signature that supports stemness and maintenance of the multilineage potential of HSPCs.

### BRD9 contributes to the activity of lineage regulatory transcription factors

**Fig. 5:**
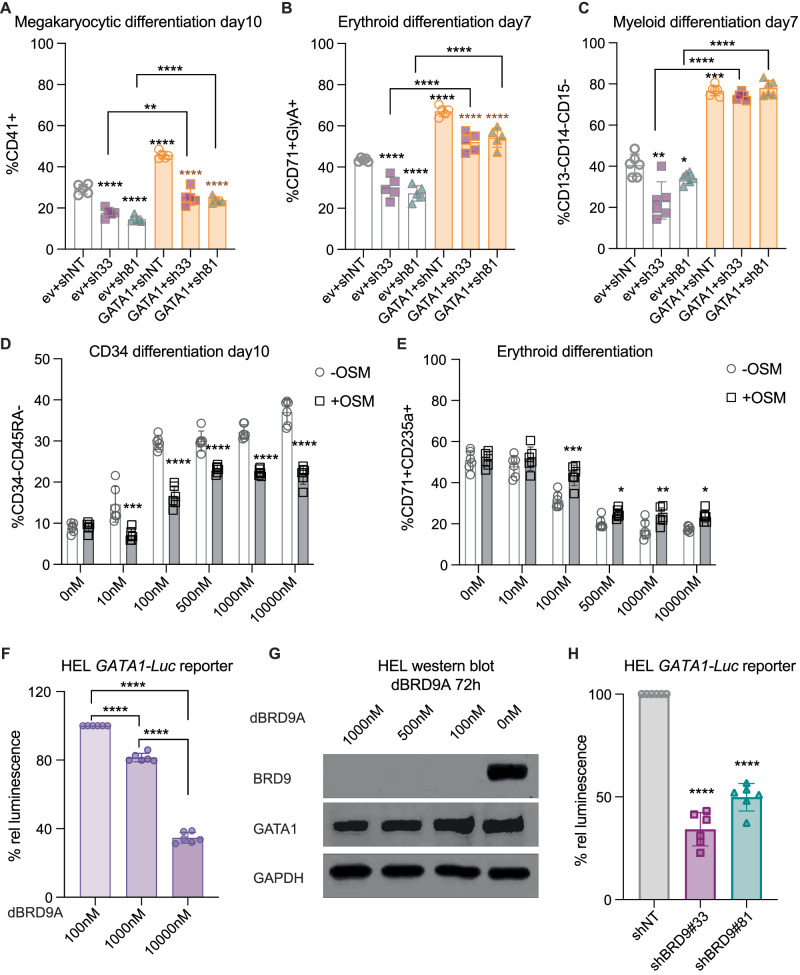
HSPC differentiation phenotype rescue and modulation of GATA1 activity. HSPCs were co-infected with control or BRD9 shRNA and empty or *GATA1* ORF vectors and placed in lineage supportive culture conditions and their differentiation by surface marker expression was observed in **A** megakaryocytic lineage by CD41 expression, **B** Erythroid lineage by expression of CD71 and CD235a, and **C** fraction of undifferentiated cells in myeloid lineage was observed for cells negative for CD13, CD14, and CD15 expression. Black asterisks without line underneath and orange asterisks represent the difference between control and BRD9 hairpins with empty vector or GATA1 OE respectively (one of the *n* = 3 biological replicates is shown with all data points, mean ± SD is plotted and *p* values from unpaired t-tests is shown by asterisks). Flow cytometric evaluation of (**D**) differentiation along CD34 and CD45RA surface expression for CD34–CD45RA-–differentiated HSPC population with dBRD9A in absence or presence of 20 ng/mL Oncostatin M. **E** Erythroid differentiation by CD71 and CD235a expression. Significant difference between +OSM and –OSM is shown using black asterisks (*n* = 3; mean ± SD with all technical replicates, and significant *p*-value in unpaired t-test from one representative experiment is shown). **F** One Glo Luciferase reporter assay readout of HEL *GATA1-Luciferase* reporter line after dBRD9A treatment where reference luminescence is set to 100 for the lowest dBRD9A concentration. **G** Protein level expression of BRD9, GATA1 and GAPDH in HEL cells 72 h after dBRD9A treatment. **H** Luciferase reporter assay on HEL *GATA1-Luc* reporter line infected with control and BRD9 shRNA.

Knock-down of BRD9 in human HSPCs altered the function of the master hematopoietic regulator GATA1 without significantly altering its transcription (Supplementary Fig. 5A). We asked if forced expression of GATA1 (validated enhanced protein expression in Supplementary Fig. 5B) can still have any effect on the phenotypes seen after BRD9 knockdown. In three independent experiments with multiple replicates, we observed that CD34 cells co-infected with GATA1 (Supplementary Fig. 5C) show an increase in differentiation toward megakaryocytic and erythroid lineage compared to control and a partial rescue from the severe decline in megakaryocyte and erythroid differentiation in the presence of shBRD9 (Fig. 5A, B). As expected, [58], forced GATA1 expression significantly inhibited terminal myeloid differentiation (Fig. 5C). Next, we asked if the addition of oncostatin-M (OSM; a known-GATA1 regulated growth factor) which was significantly downregulated with BRD9 KD, can rescue some of the phenotypes observed with dBRD9A treatment. Notably, addition of OSM even at a low concentration of 20 ng/mL to the media was able to partially rescue the rapid differentiation and proliferation loss of CD34 cells (Fig. 5D, Supplementary Fig. 5D) and loss of erythroid differentiation and proliferation in the presence of dBRD9A (Fig. 5E, Supplementary Fig. 5E). These data suggested that regulation of GATA1, and thereby its downstream targets such as OSM, might be one of several ways through which BRD9 affects stemness and multilineage potential of human HSPCs.

To examine the attenuation of GATA1 activity, we introduced a GATA1 luciferase reporter construct (*pGATA1-Luc*) into the human erythroleukemia cell line HEL, given the reported GATA1 activity in erythroid cells and capability of HEL cells to differentiate into megakaryocyte- like cells [59]. Treatment of reporter HEL cells with dBRD9A significantly reduced the luciferase signal in a dose dependent manner (Fig. 5F, Supplementary Fig. 5F). However, reduction in the luciferase signal was not proportional to the change in GATA1 protein and mRNA levels with dBRD9A treatment (Fig. 5G, Supplementary Fig. 5G). Similarly, when the reporter line was infected with a BRD9 shRNA viral vector, a twofold loss of luciferase activity was observed without a proportional loss of GATA1 protein upon BRD9 KD (Fig. 5H, Supplementary Fig. 5H), which argues against the direct transcriptional regulation of GATA1 by BRD9. A similar observation was previously made in mouse Lin-Kit+ cells where loss of BRD9 resulted in an increased CTCF signal at chromatin sites without affecting mRNA or protein level of CTCF or its downstream targets [36]. Loss of GATA1 transcription factor activity, without a significant change in overall expression of GATA1 levels, suggest that BRD9 primarily regulates GATA1 activity through its effects on chromatin.

## Discussion

While there is a significant amount of information about the function of transcription factors in the regulation of hematopoiesis and lineage differentiation, reports on the role of epigenetic regulators, especially for the members of BAF complexes are relatively scarce. Several transcription factors are known to regulate essential gene regulatory networks for the various lineage differentiation programs and are called “master regulators” [60–63]. Epigenetic factors, such as chromatin modifiers that allow the interaction of these master regulators with the functional nucleotide elements ultimately regulate gene expression and act as “gatekeepers” of hematopoiesis [15]. Epigenetic regulators often have tissue and cell type specific functions and since the expression pattern of BRD9 in human and murine hematopoietic systems is considerably different [64], we therefore focused our investigations on the human hematopoietic system only. Using chemical and genetic depletion, we showed that BRD9 plays an essential role in the fate of human hematopoietic stem cells. BRD9 maintains the LT-HSC pool and multilineage potential, promotes terminal differentiation to megakaryocytic and erythroid lineages and negatively regulates terminal myeloid differentiation. Downregulation of BRD9 resulted in decreased levels of genes involved in differentiation such as *KLF1*, *LIF* and *OSM*. Gene ontology terms such as heme biosynthesis and translation were negatively enriched in human HSPCs after BRD9 knock down. These findings are in general agreement with the previously reported observations in meeting abstracts and a recent publication on murine HSC [36, 65, 66]. While in murine hematopoietic system expression of Brd9 in LSK and HSC population is significantly lower than B cells, the knockout mice still show a pancytopenia, and myeloid lineage skewing with most severe effects on B cell development [36].

It is suggested that megakaryocytes can directly differentiate from the HSC without significant intermediate steps [67, 68]; we observed that the loss of BRD9 was profoundly detrimental to the megakaryocytic lineage indicating that BRD9 is essential for early decisions in commitment of human HSC to this lineage. Contrary to the murine system, overall transcriptional downregulation in human HSPCs suggests that BRD9 maintains the transcriptional programs required for proliferation and differentiation at least in part by regulating the function of transcription repressors. Of the many possible mechanisms, we report that some of the effect is caused by modulation of GATA1 activity, which is a known master regulator in human hematopoiesis. Many members of the bromodomain and extra terminal motif (BET) family are reported to directly interact with GATA1, including BRD2, BRD3 and BRD4 [55]. While BRD2 and BRD4 are required for GATA1 mediated erythroid maturation, BRD3 is dispensable for GATA1 function in erythroid cells despite their widespread chromatin co-occupancy and co-purification [69]. Analysis of published ChIP data from bone marrow CD34 cells [70], erythroid progenitors [71] and the erythroleukemia line K562 [72], suggested a co-occupancy of BRD9 and GATA1 at many gene loci including the beta globin cluster. Negative results from our co-immunoprecipitation experiments in HEL cells suggested no physical interaction between the two proteins. Although, acetylation dependent interaction of BRD3 with GATA1 has been reported [73], we did not attempt biochemical purification of BRD9 with acetylated GATA1 peptides. Nonetheless, reduction in a GATA1- luciferase reporter signal after BRD9 degrader treatment or knock-down suggests a direct role for BRD9 in the regulation of GATA1 activity.

An increased number of downregulated genes after BRD9 KD in HSPCs indicate an overall state of transcriptional repression in the absence of BRD9. This can be explained by three possible scenarios: (1) BRD9 hinders binding of repressive TFs to the promoters and enhancers, and loss of BRD9 makes the chromatin more accessible to the transcriptional repressors; (2) in the absence of BRD9, the multi-component assemblage required for active transcription is rendered dysfunctional; or (3) active transcription halts altogether at multiple composite elements (enhancer/ TF, promoter/TF) in the absence of BRD9. While the core ATPase component of ncBAF complex, BRG1, is known to be recruited at GATA1 containing functional composite elements [74], a recently published report on murine HSC suggests that BRD9 regulates gene expression via chromatin accessibility and looping [36], supporting the first scenario above. Our ATACseq data shows a gain in chromatin accessibility at regions containing repressive transcription factors, and one such TF, ZBTB7a, is reported to co-occupy GATA1 target regions [75]. The modulation of GATA1 activity by BRD9 through direct or indirect interaction in part seems to be a critical determinant of the phenotype observed in HSPCs after BRD9 downregulation, as shown by our studies on human HSPCs and the HEL erythroleukemic line. The exact mechanism with which this interaction plays out to regulate lineage differentiation could be a part of one or several complex regulatory circuits reported for GATA1 activity in the process of hematopoiesis and lineage differentiation [63].

In conclusion, our data suggest an essential role of BRD9 in the fate determination of human hematopoietic stem and progenitor cells. Maintenance of stem cell pool and commitment to lineage differentiation is regulated by BRD9 through modulation of master regulators of transcription. Near-complete abrogation of megakaryocytic lineage in the absence of BRD9 and accelerated terminal myeloid differentiation could theoretically be useful in managing pathologic conditions of megakaryocytic hyperactivity, such as essential thrombocythemia or other disorders associated with thrombocytosis.

### Supplementary information

Supplementary methods, figures-legends, table legends

Original uncut western blot

Supplemental table 1

Supplemental table 2

Supplemental table 3

Supplemental table 4

## Data Availability

RNAseq data in the form of DESeq2 results from each shRNA against BRD9 compared to non-target control is provided as supplemental table 2 and 3. Processed ATACseq data of merged combined peaks from shBRD9 compared to shNT is provided as supplemental table 4. The sequencing data from this study are available at GEO as GSE264008 and GSE264030. Gene expression level comparison plots were prepared from publicly available RNAseq dataset GSE97104 [76] and GSE115798 [77]. Published ChIPseq data is available at GSM970258 [70], GSM607949 [78], GSM3523237 [71] and ENCODE project [72].
