# Supplementary material for: BRD9 regulates normal human hematopoietic stem cell function and lineage differentiation
Source: Cell Death Differ. 2024 May 30;31(7):868–80. doi: 10.1038/s41418-024-01306-5 (PMC11239944; doi:10.1038/s41418-024-01306-5)
Supplement: Supplementary file 1 — Supplementary methods, figures-legends, table legends [file 41418_2024_1306_MOESM1_ESM.docx]

# Supplementary methods:

**Cell lines:**

Human AML cell line MOLM-14 (DSMZ#ACC777), SKM1 (DSMZ#ACC547), MV4-11 (ATCC#CRL9591) and human erythroleukemia cell line HEL (#ACC 11) were maintained in RPMI1640 (Gibco#61870036) supplemented with 10%FBS (Gibco #10437-028), and penicillin/streptomycin (Gibco#10378016). Transformed human embryonic kidney cell line Lentix-X 293T was purchased from Takara (#632180), maintained in DMEM (Gibco #10564011) + 10% heat inactivated FBS. Early passage cells were cryopreserved in freezing media composed of FBS and 10%DMSO (Sigma #D2438-50ML).

**Lentivirus production and infection:**

Lentiviral (LV) packaging plasmids psPAX2 (#12260) and PMD2.G (#12259) were purchased from Addgene, gifts from Didier Trono, EPFL, Switzerland. High titer lentiviral vectors were produced in confluent Lenti-X-293T cells one day after initial plating in 15cm dishes; cells were transfected using 72µL turbofect (Thermo#R0532) and a combination of 15µg psPAX2, 4.5µg PMD2.G and 7.5µg plasmid of interest prepared in OptiMEM (Gibco #31985070). Culture media was replaced with reduced 3%FBS DMEM at 16 hours post transfection. Supernatants were collected at 48 and 72 hours post transfection, passed through 0.4µm syringe filter, overlaid on 0.2µm filtered 20% sucrose in water (Fisher Scientific #S5-500) in ultracentrifuge tubes (Beckman Coulter #C14292) and spun at 29000 rpm (Beckman Coulter rotor SW 32 Ti) for 2 hours at 4℃. Lentiviral pellets were dissolved in 80µL IMDM, aliquoted and stored at -80℃ before titration on AML cell lines. AML cell lines were spin infected at multiplicity of infection (MOI) 5, using 10µg/mL polybrene (Sigma #TR-1003) at 1800 rpm for 30 min at room temperature (RT). CD34 single infections were done at MOI 30 using 10µg/mL protamine sulfate (Sigma #P4020-1G) and double infections were carried out at MOI 25 each LV with a combination of 100µg/mL poloxamer 407 (Sigma #16758-250G), and 10µM prostaglandin E2 [(39)](https://sciwheel.com/work/citation?ids=11551362&pre=&suf=&sa=0) (Tocris #S3003) with a short spin of 1200 rpm for 5min at RT. Cells were infected for 16 hours, and triple washed with complete media change for further use.

**Luciferase reporter and transfection:**

The luciferase reporter vector pGL4.20[luc2/Puro] was purchased from Promega (#E6751). Genomic DNA (gDNA) was isolated from HEL cells using DNeasy Blood and Tissue kit (Qiagen #69504) and the GATA1 promoter region was subjected to PCR amplification from this gDNA template using infusion primers for KpnI forward and BglII reverse into pGL4.20 (for oligo sequences see supplementary table1). The 1kb fragment was gel purified and ligated between KpnI (Thermo #FD0524) and BglII (Thermo #FD0083) sites in the pGL4.20 vector using a Takara In-Fusion cloning kit (#639647). NEB stable competent bacteria (#C3040H) were transformed and colonies were picked for plasmid isolation (Qiagen #27406) and validation by diagnostic digest and Sanger sequencing. Primer details for amplification, Sanger sequencing and RT-PCR are provided in supplementary table 1. HEL cells were transfected with 2µg plasmid DNA using 6µL Viafect transfection reagent (Promega#E4982) and after 48 hours of transfection, placed in selection media containing 1µg/mL puromycin (Thermo #A1113803) for three weeks. Vials of stably transfected *GATA-Luc* and empty vector-*Luc* were cryopreserved and used in assays read by the One-Glo luciferase reporter assay reagent (Promega #E8110) on a Spectramax-M3 (Molecular Devices) spectrophotometer with plate reader.

**Flow cytometry:** Flow cytometry acquisition was done on BD LSR Fortessa 4 laser, 17 parameter machines equipped with a high throughput system (HTS) device. Cell sorting was performed with the help of DFCI Flow core staff on BD Aria. Acquisition software was BD FACS-Diva and analysis software was BD-Flowjo for all the experiments. Flow cytometry data was pre-gated on FSC vs Time for uniform events and FSC-H vs FSC-A and SSC-W vs FSC-A for singlet analysis.

Proliferation analysis was done in multi-well plates after placing equal numbers of cells in the same volume per condition. At each time point half of the well volume was transferred to a round bottom plate to which 2X DAPI solution was added before readout, fresh media was replenished to the original wells. Equal well volume was acquired on HTS per condition for cell enumeration along with signal intensity from GFP or mCherry or the double-positive population. A relative change (delta) in the fraction of fluorescent positive cells was calculated from the initial time point.

Apoptosis assay was done in multi-wells by staining cells with 2X cocktail of FxCycle Violet (DAPI) (Thermo #F10347) and Annexin V (Biolegend# 640943) in binding buffer (Biolegend #422201). Cells were defined as apoptotic (Annexin V+DAPI +/-), necroptic (Annexin V-DAPI +) and live (Annexin V-DAPI-).

DNA content analysis was done in triplicate tubes from cells placed in 48 well plates. Cells were washed, formaldehyde fixed for 10 min at RT, sequentially permeabilized using pre warm 0.1%triton x-100 for 10 min at 37℃, 0.05%triton x-100 (in 1%BSA-PBS) and ice cold 70% methanol for 20 minutes on ice before washing with cold 1%BSA-PBS. Cells were stained with 10µL of 1µg/mL Pyronin y (Thermo #J61068) for 10 min at dark followed by 5uL of FxCycle violet for next 20 minutes before 2x wash with 1%BSA-PBS followed by the acquisition. Pyronin low (RNA) and DAPI (DNA) low fraction was defined as G0/subG1, pyronin positive and DAPI low (n) population as G1, pyronin positive and DAPI high (4n) as G2M and cell fraction with intermediate DNA staining was defined to be in S phase.

Differentiation was measured by cell surface immunophenotype as the output of cells under genetic or chemical selection in optimal CD34 media, specialized HemaTox media or in the cells recovered from *in vivo* growth in immunocompromised mice. All the fluorochrome labeled antibodies were purchased from Biolegend unless mentioned otherwise and TruStain Fc block (#422302) was used before addition of antibodies. CD34-APC (#555824), and CD45RA-PE/Cy7(#304126) were used for initial differentiation assay in CD34 optimal media and for FACS sorting in lineage-priming media described in figure 2. Megakaryocytic differentiation was assessed using CD41-Bv421 (303730), erythroid with CD71-Bv650 (#334116) and CD235a-APC (GlyA #551336), and myeloid differentiation using CD13-Bv711(#301722), CD14-APC/Cy7 (#325620), CD15-PE (#323006) for the cells in HemaTox media. Cells harvested from mouse bone marrow were stained with CD45-Bv785 (#304048) to assess human engraftment, and output into lymphoid with CD19-PE/Cy7 (#302216), CD3-Bv711 (100241), myeloid with CD33-PE (303403), erythroid with CD71-Bv650, CD235a-perCP (349110), megakaryocytic with CD41-Bv421 and into stem pool with CD34-APC were assessed after short, intermediate and long-term studies in mice. Single color compensations were carried out using UltraComp beads (Thermo #01-2222-42) for all panels.

**Western Blot:**

Primary cells were washed twice in PBS before direct lysis with sample buffer (Bio Rad #1610747) containing 10% (v.v) 2-mercaptoethanol (Bio Rad #1610710) and boiled at 100℃ for 10 minutes. Lysates were loaded onto precast gels (Bio Rad # 4561095) and proteins were transferred onto nitrocellulose membranes (Bio Rad #1620097). Membranes were blocked with 5% (v/v) non-fat milk in TBST for 1hr at RT before overnight incubation with primary antibodies. Primary antibodies against BRD9 (# 58906), GATA1 (#3535), and GAPDH (#2118) were purchased from Cell Signaling Technologies, anti-FLAG antibody was used to detect FLAG-tagged BRD9 (Sigma #F1804-200UG). HRP-conjugated anti-mouse (#NA-935) and anti-rabbit (NA-934) secondary antibodies were purchased from Cytiva. Blots were imaged using ECL substrate (Bio Rad #1705062).

**RNAseq and RT-PCR:**

Cells were washed in cold PBS and pellets were resuspended into Trizol reagent (Thermo #15596026) before RNA extraction using RNeasy kit (Qiagen #74104). RNAseq was performed commercially through Azenta Life Sciences. Samples were quantified on Qubit fluorometer, and RNA integrity was checked on Agilent tapestation. Libraries were prepared using NEB Nextra II library prep kit for Illumina using oligo(dt) bead-based enrichment followed by first and second strand synthesis. Sequencing libraries were quantified and validated before clustering on flow cells which were loaded onto Illumina HiSeq instrument and sequenced using 2x150bp paired end configuration. Raw sequences were converted into fastq, indexed, de-multiplexed and trimmed before mapping to human genome using STAR aligner. BAM files were generated, and unique gene hit counts were calculated from Subread package v.1.5.2 which were used in downstream differential expression analysis using DESeq2 where Wald test was used to generate p-values and Log2 fold change. For RT-PCR, RNA samples were quantified on the NanoDrop Spectrophotometer. Equal amount of RNA was incubated with oligo(dT) and dNTPs (Promega #U1515) at 65℃ for 5 min and rapidly chilled before cDNA synthesis using M-MLV RT kit (Thermo #28025013). Synthesized cDNA samples were diluted 1:3 with ultrapure water before downstream application in RT-PCR using SYBR green master mix (Thermo #A25776).

**ATACseq:**

Cells were prepared in triplicates, FACS sorted, washed with PBS, treated with DNAse (Worthigton #LS002006) and cryopreserved with FBS+10%DMSO. ATACseq was done commercially through Azenta Life Sciences where cells were thawed, washed and DNase treated before cytosol removal from live cells. Nuclei were treated with Tn5 enzyme (Illumina #20034197) for 30 min at 37℃ and purified with Minelute PCR purification kit (Qiagen #28004). Tagmented DNA was barcoded with Nextera index kit v2 and PCR amplified prior to SPRI selection to yield DNA libraries which were clustered on a flow cell and loaded on the Illumina HiSeq instrument. The samples were sequenced 2x150bp PE configuration and raw files were converted into fastq. After initial quality check and trimming, clean reads were aligned to hg38 using bowtie 2 and filtered using samtools. Mitochondrial reads and unplaced contigs were called and filtered. MACS2 2.1.2 was used for peak calling to identify open chromatin regions. Valid peaks from each sample per group were merged and peaks called in at least 66% of samples were kept for downstream analysis and used in differential peak analysis using the R package DiffBind.

# Supplementary figure legends:

Sup. Fig.1: (A) pLKO.1shRNA vector design and protein level knockdown in HSPC sorted for GFP+ fraction 5 days post infection, expanded in myeloid primed culture condition and taken for lysate. (B) BRD9 overexpression vector design with shBRD9#81 target site in wild type ORF and introduced silent mutations in hairpin resistant mutant shown in red, western blot showing protein level rescue in GFP+mCherry+ co infected MOLM 14 cell line. (C) Comparison of *BRD9* expression levels in GSE115798 shows highest expression in undifferentiated HSC compared to lineage restricted cells: hematopoietic stem cells (HSC), lineage restricted progenitors (CD49f+), multipotent progenitor (MPP), lymphoid primed multipotent progenitors (LMPP), and multi lymphoid progenitors (MLP) (Individual points with mean values are plotted, p values from ordinary ANOVA with Tukey's multiple comparison test are shown only for comparison between HSC and other populations). (D) *BRD9* expression in cultured CBCD34 using RT-PCR (n=2 biological replicates, mean and SD are plotted, and individual data points are shown). (E-F) DNA content profile using pyronin gamma and DAPI on GFP+ HSPCs is shown for one representative experiment (n=3) by flow cytometry plots (one of 4 technical replicates) and mean and SD along with individual data points are plotted, p values in two-way ANOVA with Dunnett's multiple comparison test compared to control shRNA is shown­. (G) Proliferation rescue in MOLM14 cells with BRD9 ectopic expression in combination with hairpin infection (mean and SEM for one representative of n=3 independent experiments is plotted and p values from unpaired t-test is shown). (H) Increase in CD34 negative population at a single time point in four different shRNA against BRD9 (n=3, mean and SEM from one representative experiment and p values from unpaired t-test compared to control are shown). (I) Dose dependent differentiation of CD34 cells to CD34-CD45RA- with dBRD9A treatment (n=4, individual data points with mean and SEM from one biological replicate and p values compared to control in ordinary ANOVA with Tukey’s multiple comparison test are shown).

Sup. Fig.2: (A) White field merged images of day 12 readout of methylcellulose colony formation assay performed using equal number of GFP+ cells in each condition per well, cells with hemoglobin content which are red under bright field microscope are seen as dark gray on BioRad chemidoc white field. (B) Methylcellulose CFC assay in presence of DMSO and increasing concentration of Novartis BRD9 degrader QA68 (n=2, mean and SD are plotted) [(26)](https://sciwheel.com/work/citation?ids=15411975&pre=&suf=&sa=0&dbf=0). Cell proliferation, surface marker expression on differentiated cells and Giemsa stained cytospin images in 60x oil immersion of HSPCs in presence of DMSO and dBRD9A cultured in (C) megakaryocytic, (D) erythroid and (E) myeloid priming media (individual data points with mean and SEM from one of the n=5 biological replicates are plotted, p values only for difference between DMSO and dBRD9A concentration are shown from ordinary one way ANOVA with Dunnett's multiple comparison test).

Sup. Fig.3: (A) Representative flow cytometry plots of GFP and human CD45 expression in mice bone marrow at week 16 in one mouse each that received CD34 cells infected with control hairpin (shNT n=7), shBRD9#33 (n=6) and shBRD9#81 (n=6) . (B) Total human CD45 engraftment per group that depicts fitness of injected cells per condition. (C) Lymphoid to myeloid population ratio showing no difference or selection bias for in-vivo engrafted cells. (D) Fraction of CD41+ megakaryocytic and (E) CD71+ erythroid population in engrafted GFP+ humanCD45+ cells. Plots show median value as the horizontal line; y-axis is in the log-scale; difference between control and BRD9 KD conditions is calculated by unpaired t-test and the significance is shown by depiction of p values.

Sup. Fig.4: (A) Experimental layout for RNAseq and ATACseq analysis in infected CBCD34 cells at 72 hours post infection, GFP+ sorted cells. (B) Volcano plot showing differential gene expression analysis with shBRD9#81 compared to non-target control hairpin. (C) TF enrichment analysis on common downregulated genes with the 2 shBRD9 hairpins when queried against CHEA dataset. (D) Heatmap showing expression of significantly different transcription cofactors (TcoF). (E) Gene ontology enrichment analysis for biological processes on differentially accessible genes in ATACseq. (F-I) IGV ChIP tracks showing GATA1 and BRD9 interaction with chromatin region (fold change over control) at KLF1, MXD3, beta globin cluster and HOXB cluster.

Sup. Fig.5: (A) *GATA1* expression in GFP+ CD34 cells 72 hours post infection with control or shRNA vectors against BRD9 using RT-PCR (n=3; mean±SD). (B) Schematic of *GATA1* ORF vector and protein level validation of GATA1 in infected MOLM14 cell line. (C) Flow cytometry example plots showing fraction of GFP and mCherry co-positive fraction after shRNA and GATA1 OE vector co-infection from one representative experiment. (D) Proliferation of HSPCs in CD34 optimal media and (E) Erythroid supporting media in the absence or presence of 20ng/mL Oncostatin-M in presence of increasing concentration of dBRD9A (n=3; one representative experiment showing mean±SD and all data points). (F) One glo luciferase reporter assay on HEL cells stably transfected with empty pGL4.2-Luc or GATA1 promoter (pGATA1)-Luc show increased luciferase activity in GATA1 reporter line which is reduced after dBRD9A treatment. (G) mRNA level expression of GATA1 in HEL cells treated with increasing concentrations of dBRD9A. (H) Protein level expression by western blot in HEL *GATA1-Luc* reporter line infected with control and BRD9 shRNA.


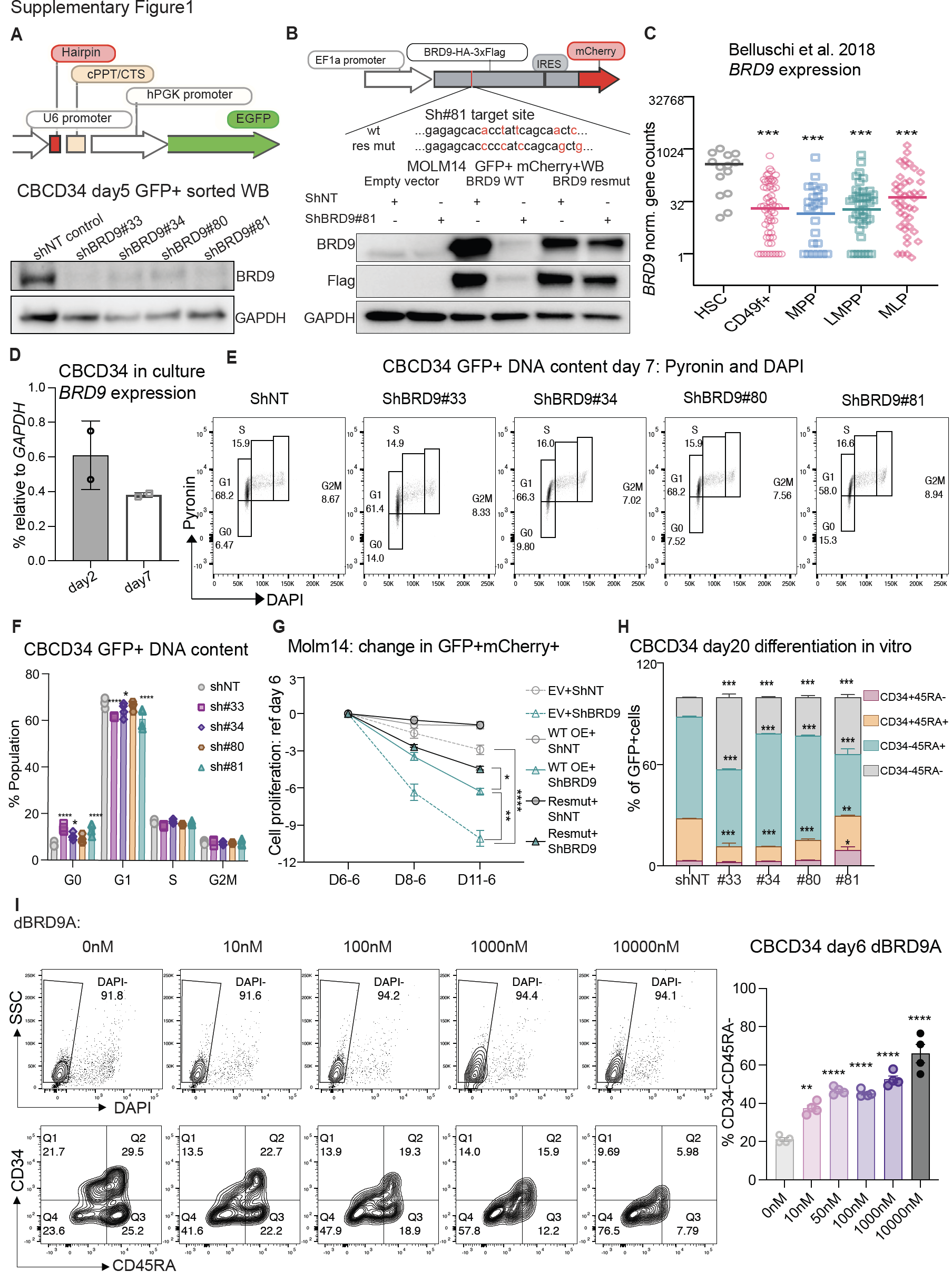


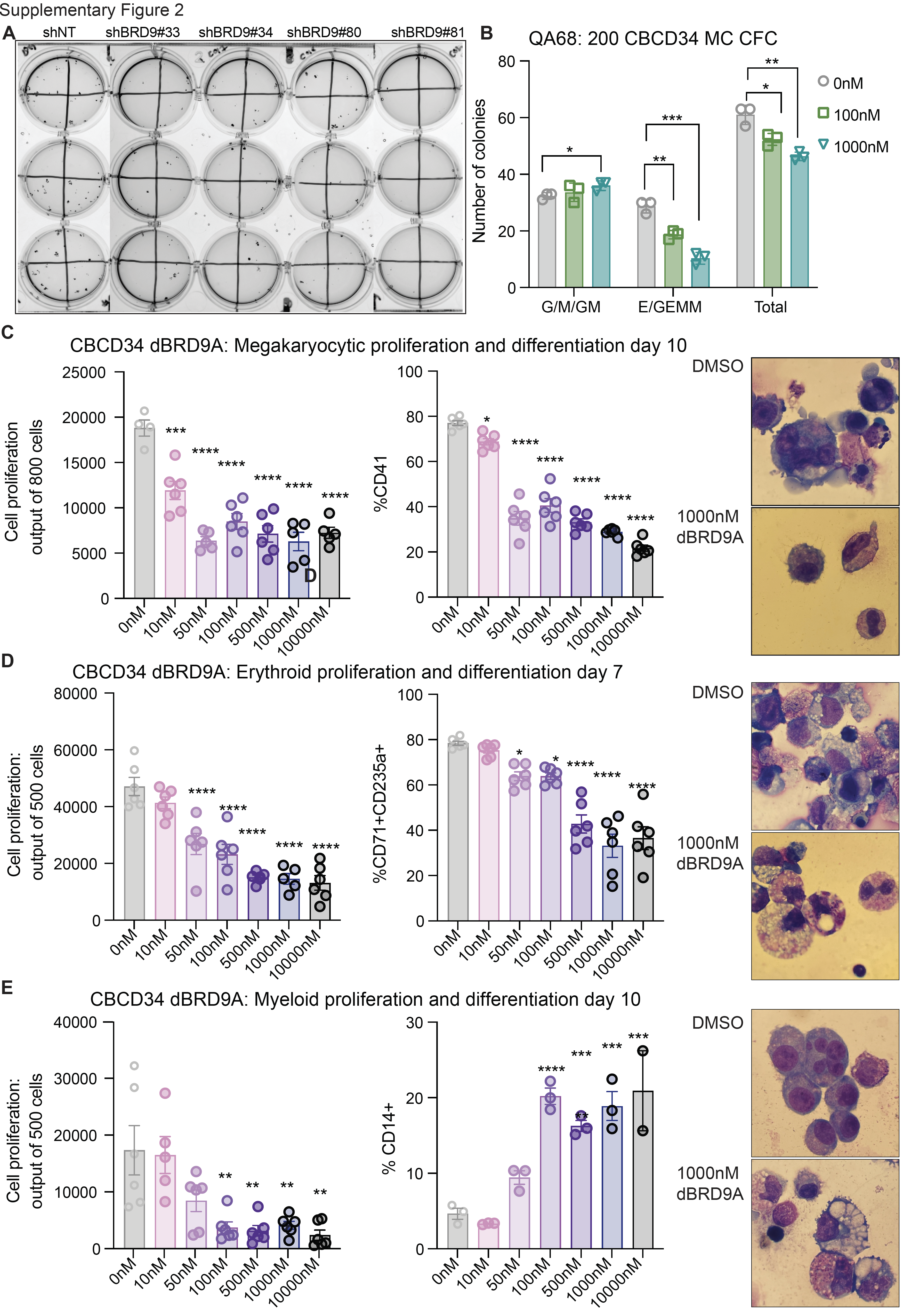


**
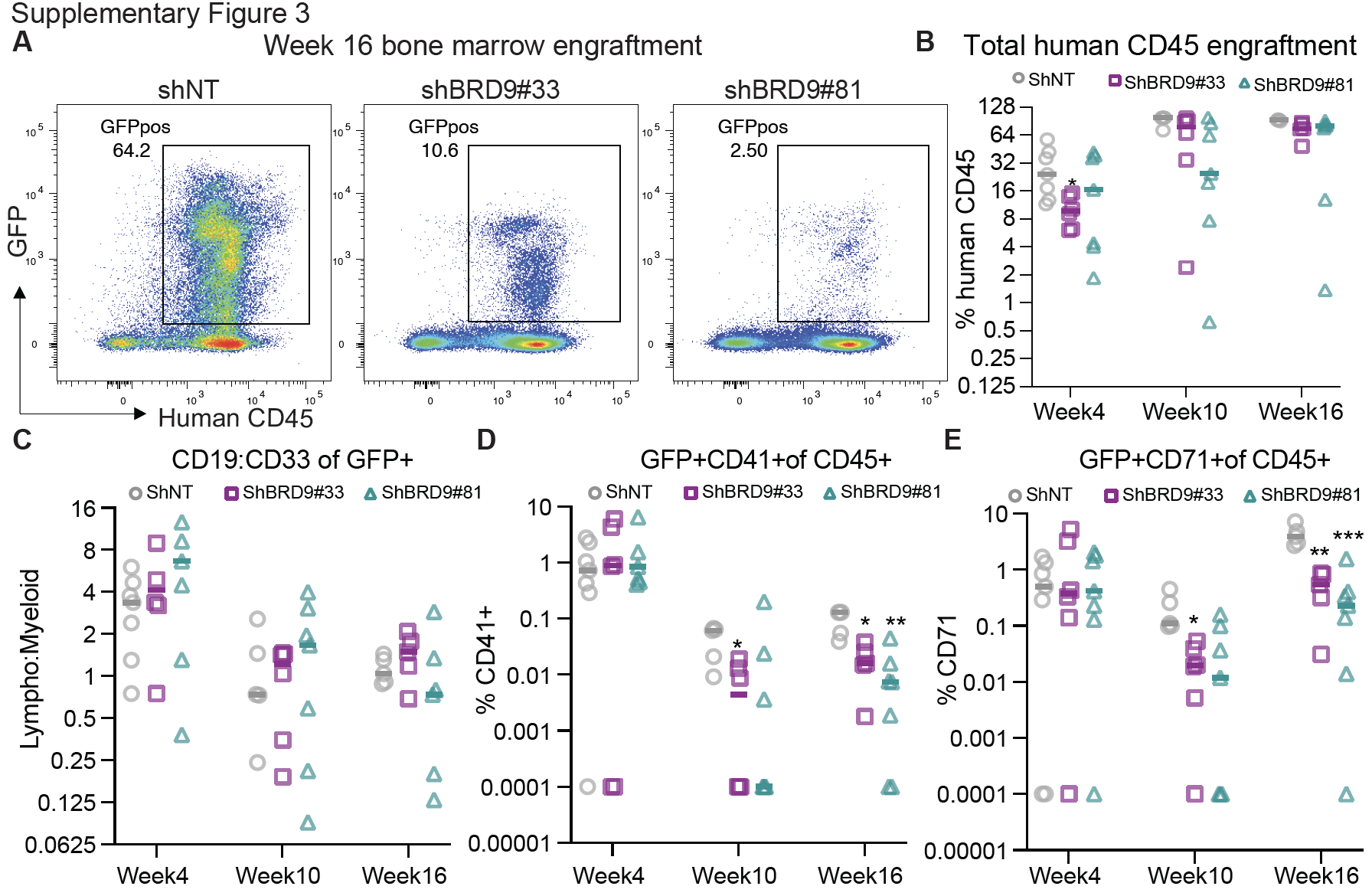
**


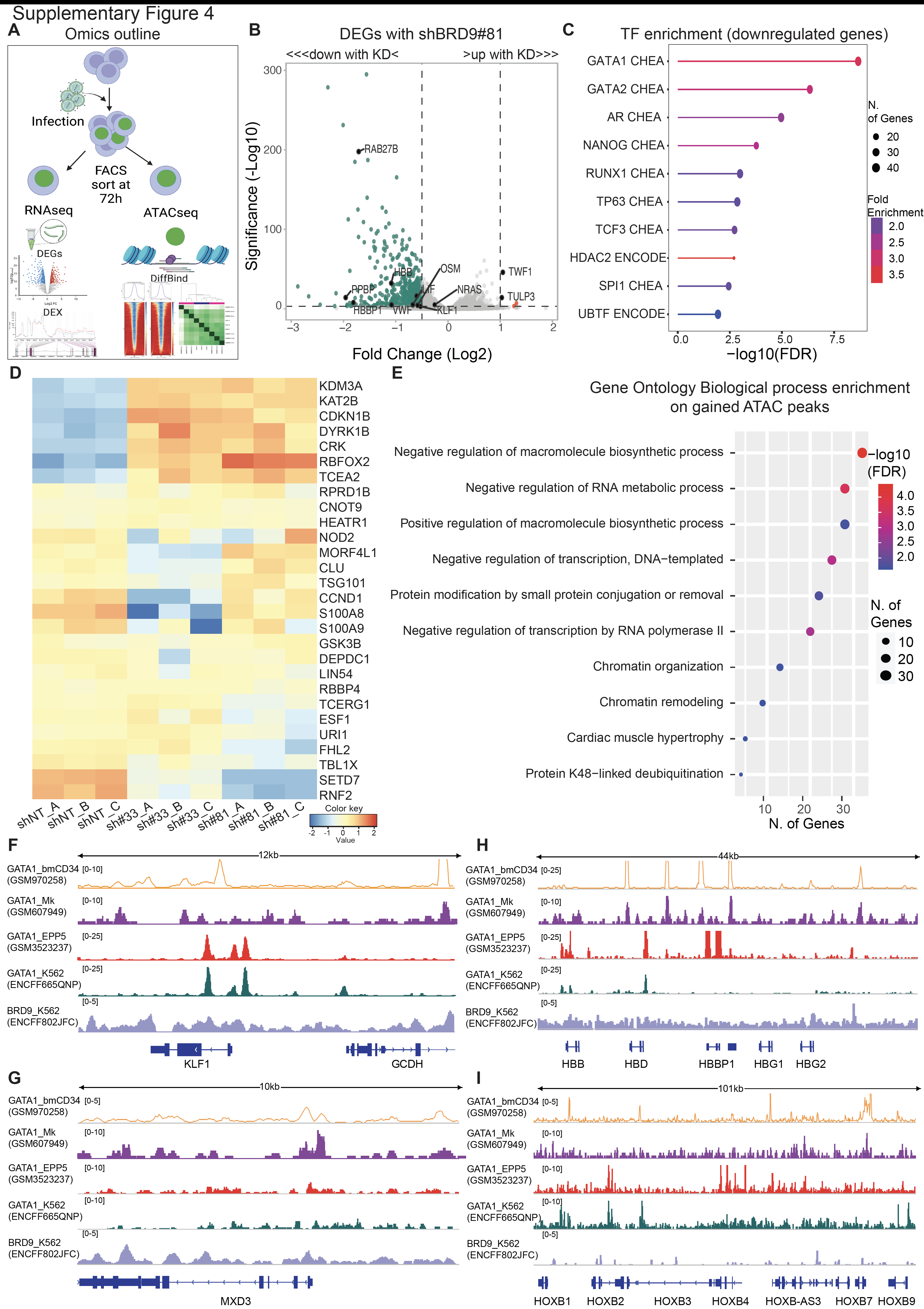


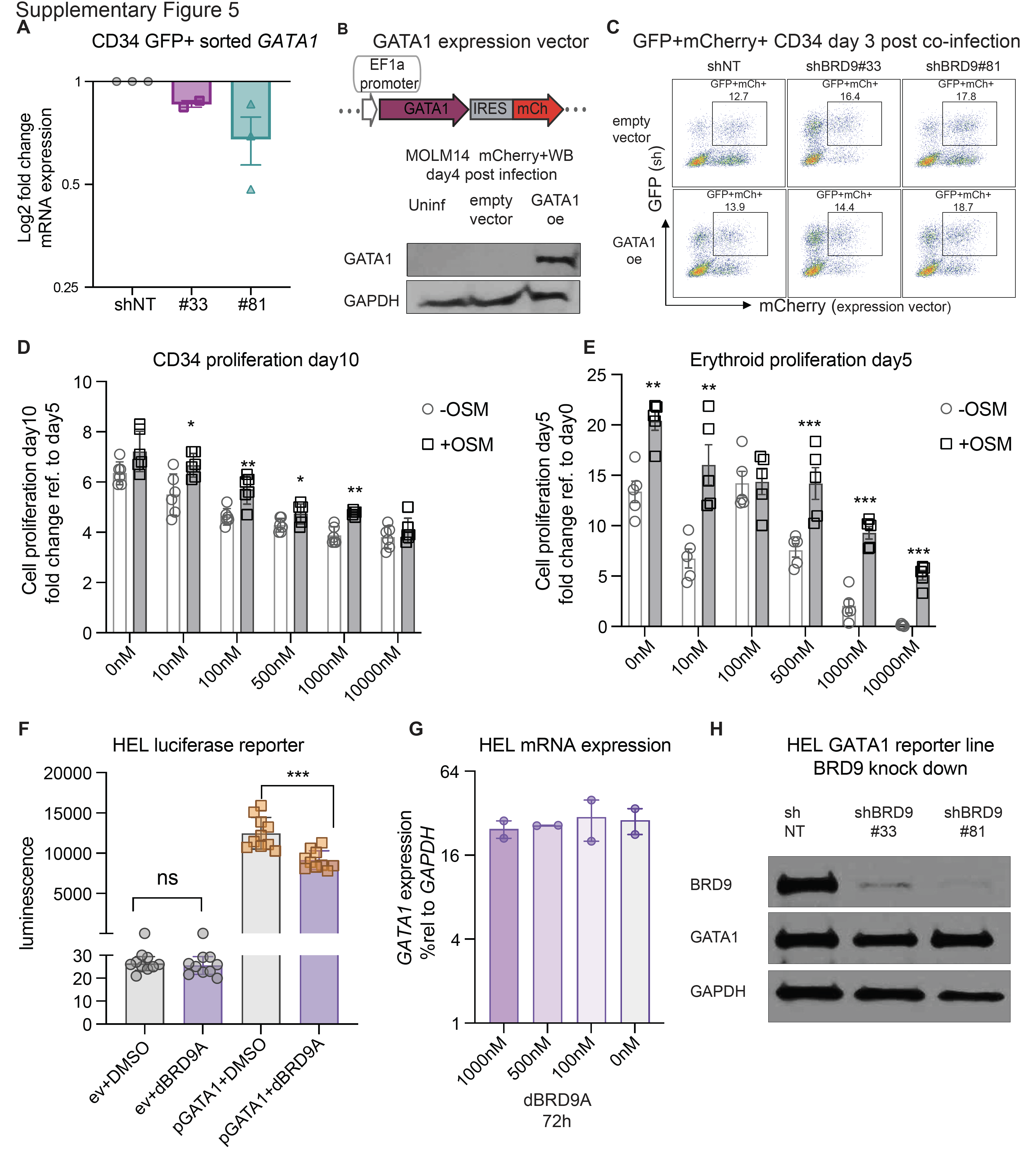


Supplementary table legends:

Supplementary table 1: Oligonucleotide sequence names and designs

Supplementary table 2: RNAseq data Deseq2 analysis of control vs shBRD9#33

Supplementary table 3: RNAseq data Deseq2 analysis of control vs shBRD9#81

Supplementary table 4: ATACseq data DiffBind analysis for control vs knock down (merged shBRD9#33 and shBRD9#81)
