# Supplementary material for: BRD9 regulates normal human hematopoietic stem cell function and lineage differentiation
Source: Cell Death Differ. 2024 May 30;31(7):868–80. doi: 10.1038/s41418-024-01306-5 (PMC11239944; doi:10.1038/s41418-024-01306-5)

Original uncut western blot for CBCD34 sorted day5 on GFP+

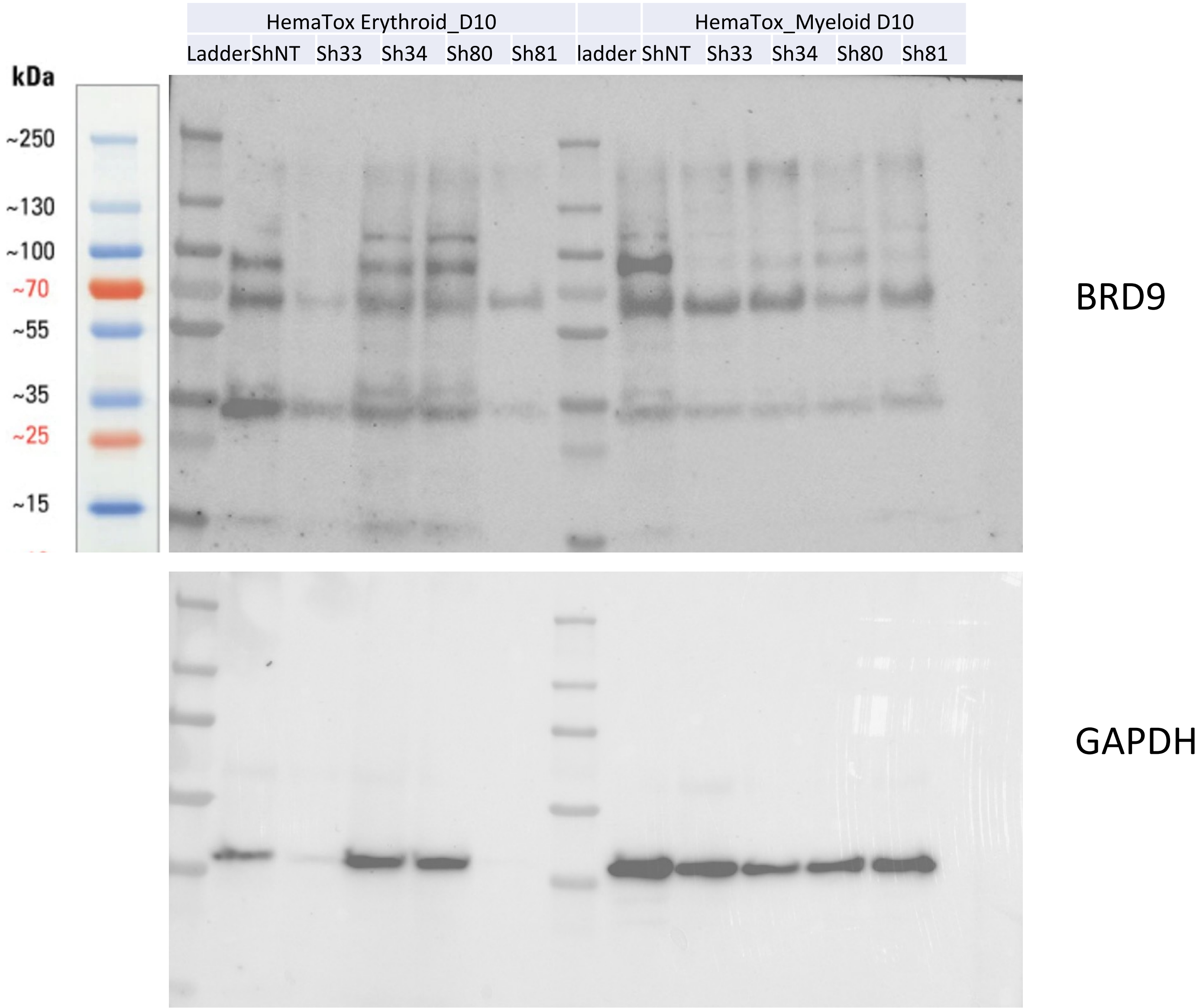

Original uncut western blot for Molm14 sorted GFP+mCherry+

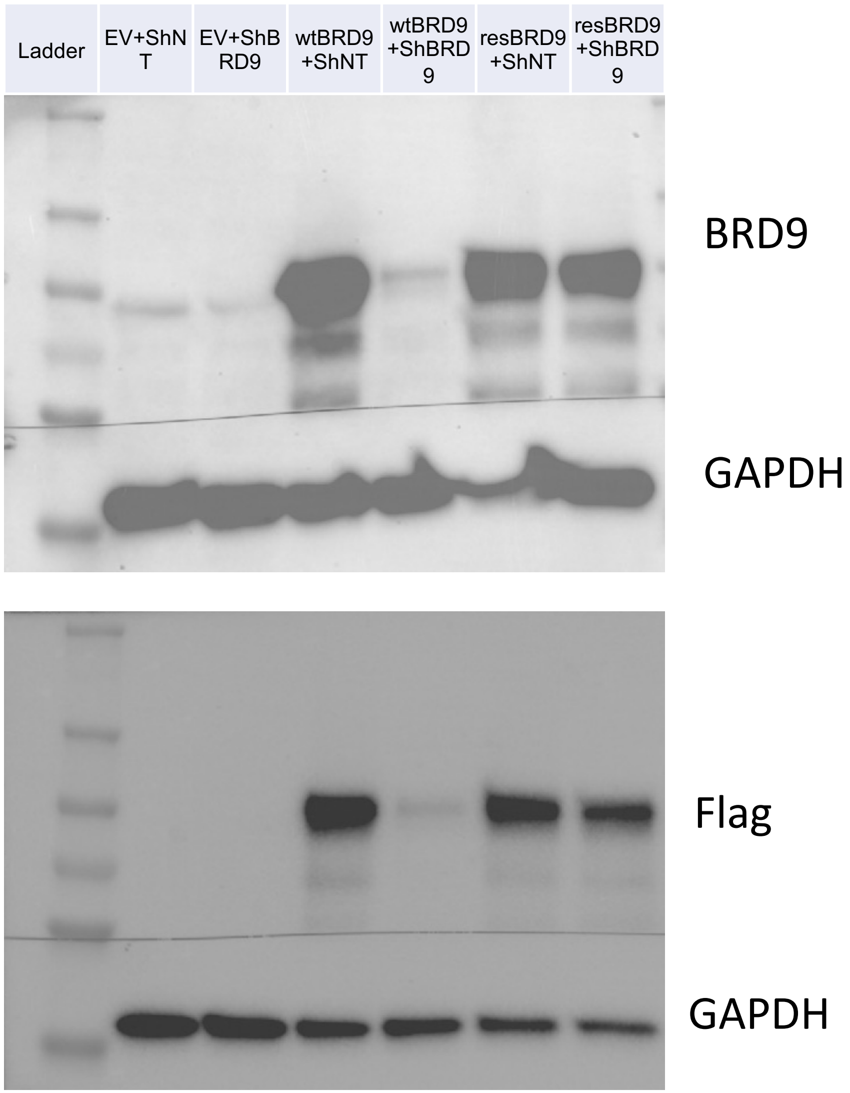

Original uncut western blot for HEL *GATA1-Luc* reporter: infected with BRD9 hairpins

BRD9

ladder	Uninfected	shNT	shBRD9 #33	shBRD#81

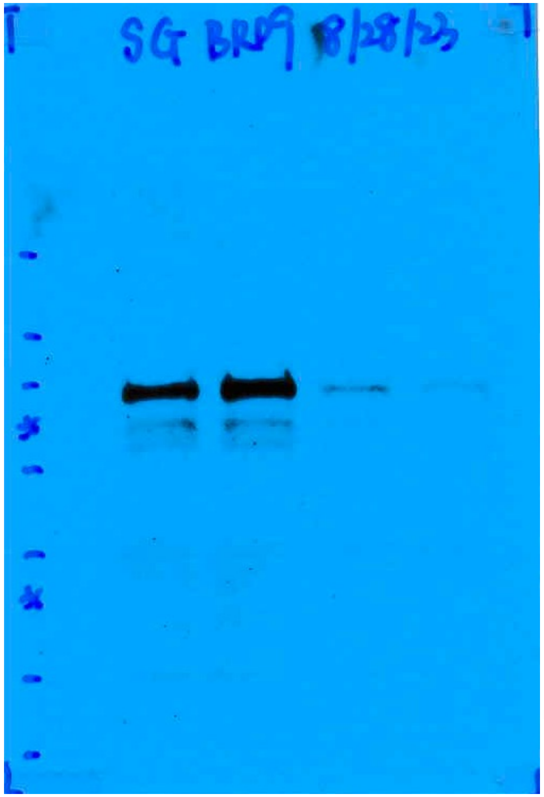

GATA1

ladder	Uninfected	shNT	shBRD9 #33	shBRD#81

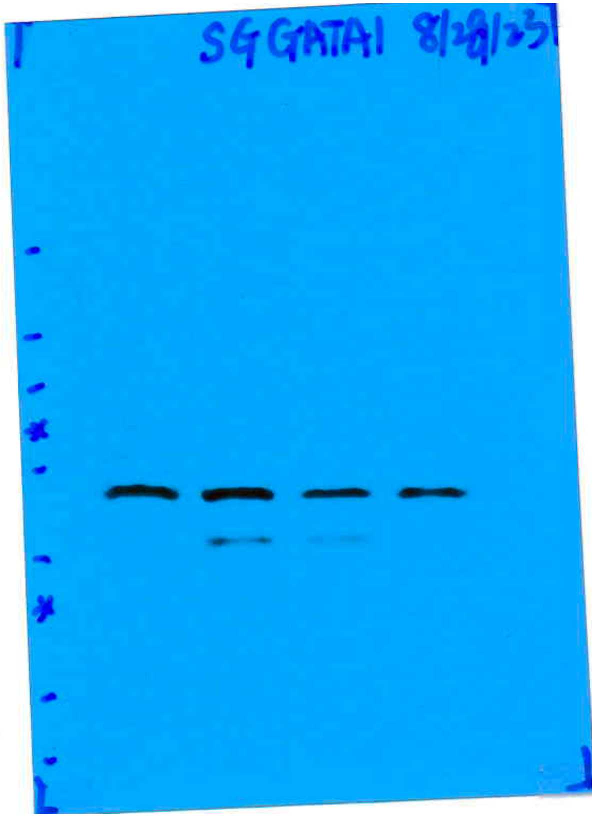

GAPDH

ladder	Uninfected	shNT	shBRD9 #33	shBRD#81

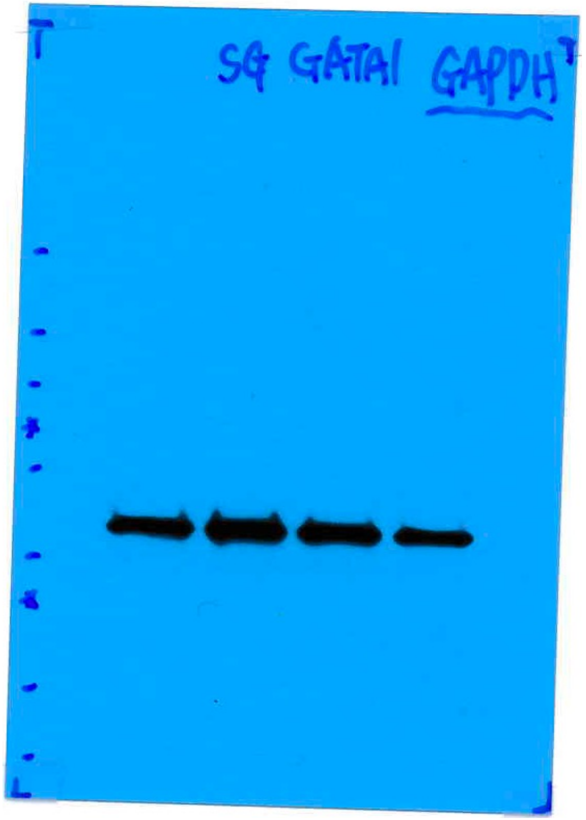

Original uncut western blot for HEL treated with dBRD9A

Ladder	Control	1000 nM	500nM	100nM	0nM	Ladder	Control	1000 nM	500nM	100nM	0nM

Ladder	Control	1000 nM	500nM	100nM	0nM	Ladder	Control	1000 nM	500nM	100nM	0nM

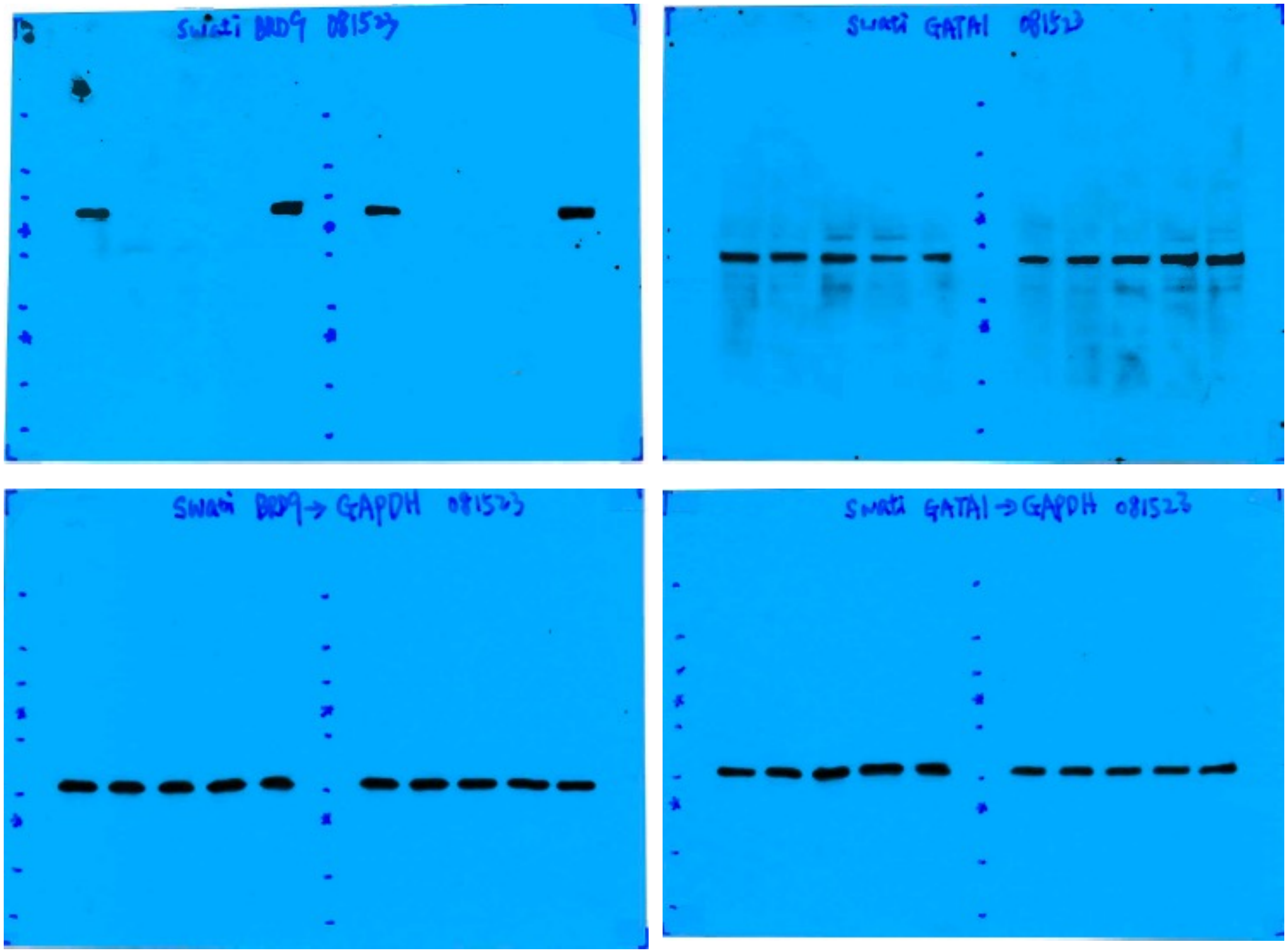

BRD9

GATA1

GAPDH

GAPDH

Original uncut western blot for MOLM14 GATA1 over expression vector validation

GATA1

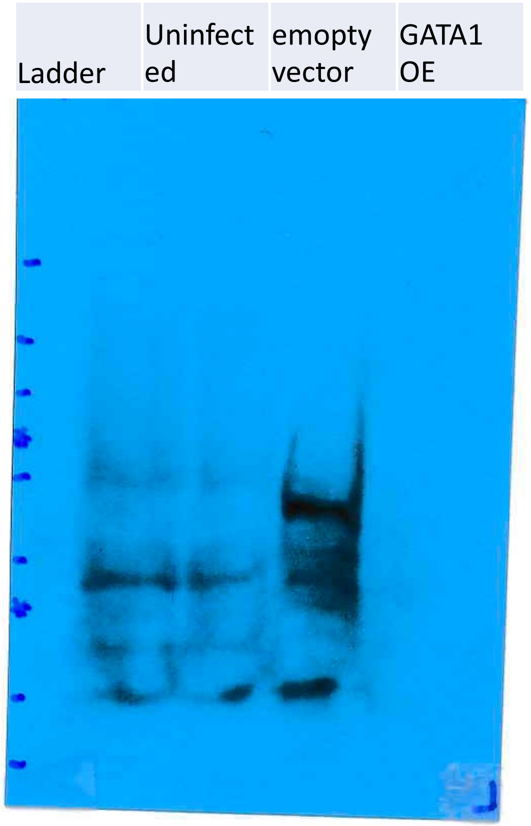

GAPDH

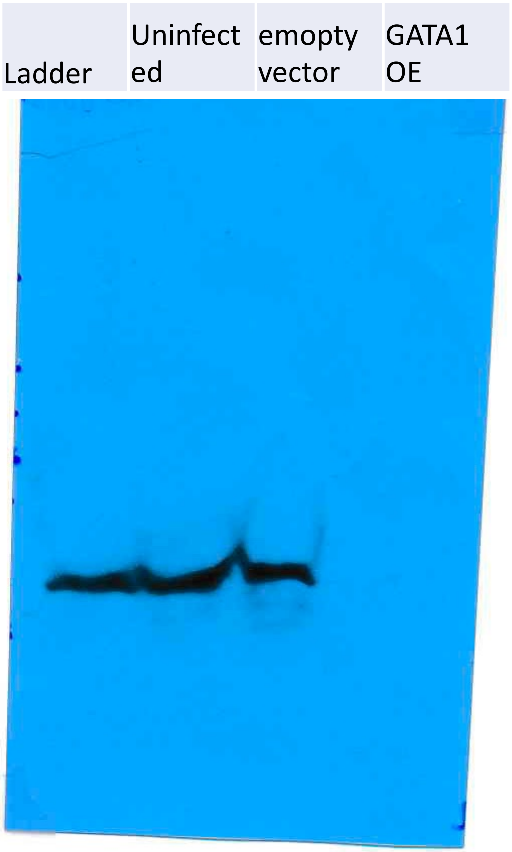

Supplement: Supplementary file 2 — Original uncut western blot [file 41418_2024_1306_MOESM2_ESM.pdf]
